# Predicting *In Vivo* Anti-Hepatofibrotic Drug Efficacy Based on *In Vitro* High-Content Analysis

**DOI:** 10.1371/journal.pone.0026230

**Published:** 2011-11-02

**Authors:** Baixue Zheng, Looling Tan, Xuejun Mo, Weimiao Yu, Yan Wang, Lisa Tucker-Kellogg, Roy E. Welsch, Peter T. C. So, Hanry Yu

**Affiliations:** 1 Computation and Systems Biology Program, Singapore-MIT Alliance, National University of Singapore, Singapore, Singapore; 2 Institute of Bioengineering and Nanotechnology, A*STAR, Singapore, Singapore; 3 Mechanobiology Institute, National University of Singapore, Singapore, Singapore; 4 Singapore-MIT Alliance for Research and Technology, BioSyM, Singapore, Singapore; 5 Department of Physiology, Yong Loo Lin School of Medicine, National University of Singapore, Singapore, Singapore; 6 Department of Chemistry, Faculty of Science, National University of Singapore, Singapore, Singapore; 7 NUS Graduate School for Integrative Sciences, National University of Singapore, Singapore, Singapore; 8 NUS Tissue-Engineering Programme, National University of Singapore, Singapore, Singapore; 9 Imaging Informatics Division, Bioinformatics Institute, A*STAR, Singapore, Singapore; 10 Department of Hepatobiliary Surgery, Southern Medical University Affiliated Zhujiang Hospital, Guangzhou, China; 11 Central Imaging Facility, Institute of Molecular and Cell Biology, A*STAR, Singapore, Singapore; 12 Department of Mechanical Engineering and Biological Engineering, Massachusetts Institute of Technology, Cambridge, Massachusetts, United States of America; 13 Engineering Systems Division, Sloan School of Management, Massachusetts Institute of Technology, Cambridge, Massachusetts, United States of America; Biological Research Center of the Hungarian Academy of Sciences, Hungary

## Abstract

**Background/Aims:**

Many anti-fibrotic drugs with high *in vitro* efficacies fail to produce significant effects *in vivo*. The aim of this work is to use a statistical approach to design a numerical predictor that correlates better with *in vivo* outcomes.

**Methods:**

High-content analysis (HCA) was performed with 49 drugs on hepatic stellate cells (HSCs) LX-2 stained with 10 fibrotic markers. ∼0.3 billion feature values from all cells in >150,000 images were quantified to reflect the drug effects. A systematic literature search on the *in vivo* effects of all 49 drugs on hepatofibrotic rats yields 28 papers with histological scores. The *in vivo* and *in vitro* datasets were used to compute a single efficacy predictor (E*_predict_*).

**Results:**

We used *in vivo* data from one context (CCl_4_ rats with drug treatments) to optimize the computation of E*_predict_*. This optimized relationship was independently validated using *in vivo* data from two different contexts (treatment of DMN rats and prevention of CCl_4_ induction). A linear *in vitro*-*in vivo* correlation was consistently observed in all the three contexts. We used E*_predict_* values to cluster drugs according to efficacy; and found that high-efficacy drugs tended to target proliferation, apoptosis and contractility of HSCs.

**Conclusions:**

The E*_predict_* statistic, based on a prioritized combination of *in vitro* features, provides a better correlation between *in vitro* and *in vivo* drug response than any of the traditional *in vitro* markers considered.

## Introduction

Liver fibrosis, a disease of excessive extracellular matrix (ECM) accumulation, is a common downstream response to repeated liver injury, caused by factors such as hepatitis B or C virus infection, excessive alcohol consumption, non-alcoholic steatohepatitis (NASH), autoimmune hepatitis, or drugs and toxins such as azathioprine [1], D-galactosamine [2] or low doses of paracetamol [3]. In current clinical practice, the most effective anti-fibrotic treatment is indirect: to target the underlying cause(s) of injury, as removal of primary insults may lead to spontaneous regression of fibrosis. For example, lamivudine, which blocks hepatitis B virus replication, can result in fibrosis resolution [4]. However, fully activated hepatic stellate cells (HSCs), besides being a major source of fibrotic ECM [5], also secrete a broad range of chemokines and cytokines for self-perpetuating fibrosis in the absence of primary insults [6]. As a result, indirect treatment by removing the underlying irritant is not effective in a significant population of liver fibrosis patients.

Current drug discovery efforts for direct anti-fibrotic therapies have primarily targeted activated HSCs. Over recent years, the focus in drug discovery research has shifted from cell-free approaches based on molecular targets, to cell-based systems-biology based approaches, in an effort to increase success rates and reduce the overhead costs of drug development [7]. Since multiple complex pathways are involved in fibrogenesis, it is important to study the anti-fibrotic effects of a drug in the cellular context. Several high-throughput *in vitro* screenings have been performed previously on HSCs or fibroblast cells. Xu *et. al.* (2007) established a quantitative screening platform based on TGF-*β*1 dependent fibroblast nodule formation [8]. Using this system, 8 out of 21 herbal extracts were found to have anti-fibrotic activities [9]. In other studies, HSC proliferation and apoptosis were used to assess the direct effects of drugs on HSC [10], [11]. Collagen expression is another indicator commonly used in high-throughput systems [12], [13]. These studies together with conventional low-throughput *in vitro* and *in vivo* studies have identified a diverse group of positive chemicals. The most promising ones, such as losartan, pioglitazone and Fuzheng Huayu tablets, have entered phase IV clinical trials [14].

Despite numerous efforts in anti-fibrotic drug discovery, there is no anti-fibrotic drug approved by the U.S. Food and Drug Administration. Many candidate drugs for fibrosis have failed in preclinical or clinical trials. One of the reasons is that *in vitro* data have poor correlation with *in vivo* drug effects due to the complicated pathophysiological background of hepatic fibrogenesis. As a result, drugs with high *in vitro* efficacies based on simple biochemical assays may fail to produce significant *in vivo* effects [15]. Despite the different levels of complexity between the *in vitro* and *in vivo* systems, previous studies from other fields such as drug dissolution [16], [17], have demonstrated that optimized design of *in vitro* systems can result in better correlation with *in vivo* data [18], [19].

In the present study, we quantitatively assessed and compared end-point anti-fibrotic drug responses from *in vitro* and *in vivo* models. A high-content analysis (HCA) system was established that provides a strong positive correlation with the *in vivo* drug responses. A drug efficacy predictor (E*_predict_*) was computed and optimized to have a high positive correlation with the *in vivo* drug efficacy (E*_in vivo_*) extracted from studies using rat carbon tetrachloride (CCl_4_) treatment models. This positive correlation was validated with two additional validation datasets from rat CCl_4_ preventive and dimethylnitrosamine (DMN) treatment models. Moreover, a linear *in vitro*-*in vivo* relationship was consistently observed in all three datasets, suggesting that the E*_predict_* value can also be used to rank drug efficacy and generate predictions. Drugs with higher E*_predict_* were observed to exert their primary effects by targeting HSC proliferation, apoptosis or contractility, which are consistent with previous anti-fibrosis strategies.

## Materials and Methods

### Cell culture

The human HSC cell line LX-2 was obtained as a generous gift from Dr. Scott Friedman (Mount Sinai Hospital, NY). The cells were cultured in Dulbecco's modified eagle medium with 1000 mg/L glucose (Biopolis Shared Facilities, Singapore) and 10% heat inactivated fetal bovine serum (Gibco, Grand Island, NY, USA) and incubated in 37°C in a humidified atmosphere with 95% air/5% carbon dioxide.

### Drug preparation

45 anti-fibrotic drugs and 4 non-specific control compounds not related to fibrosis were included in this study. The stock solution of each drug was prepared by dissolving the drug in dimethyl sulfoxide (Sigma-Aldrich, St Louis, MO, USA) at the maximum solubility of a drug unless the solvent is specifically indicated in the manufacturer's information sheet. The highest working concentration of each drug was determined as the IC_50_ value from a cell viability assay (Table S1) and was dispensed in the second column of a 96-well plate (Nunc, Roskilde, Danmark). 10 other working concentrations were prepared by 2-fold serial dilution from the highest concentration in the same 96-well plate from column 3 to column 12. The first column of each plate was used as a drug-free control column.

### Drug treatment

LX-2 cells were seeded in 96-well glass-bottom optical plates (Matrical bioscience, Spokane, Washington). The seeding density was 0.007 million in 100 µl medium per well, allowing cells to reach 70% confluence after a 3-day incubation. 24 hours after cell seeding, the culture medium was removed and fresh medium with drug was added and the cells were further incubated for 48 hours before the viability assay or staining was performed.

### Cell viability assay

Cell viability was evaluated using 3-(4,5-dimethylthiazol-2-yl)-5-(3-carboxymethoxyphenyl)2-(4-sulfophenyl)-2H-tetrazolium (MTS), according to the manufacturer's instructions (CellTiter 96 Aqueous One Solution Cell Proliferation Assay, Promega). MTS reagent was prepared by mixing minimum essential medium (Gibco, Grand Island, NY, USA), FBS and CellTiter One solution at a ratio of 9∶1∶2 just before the assay. 120 µl of the prepared reagent was added to each well and the plates incubated for 60 minutes in a 37°C incubator. At the end of the incubation, 100 µl of the medium was transferred to a new 96-well plate and the absorbance read at 490 nm. All readings were corrected with blank controls (MTS reagent incubated for 1 hour in 37°C in empty wells). All conditions were duplicated per experiment and all experiments were performed twice. The average values were used to determine the IC_50_ values and the highest drug working concentrations were set to be close to the IC_50_ values.

### Cell staining

Ten markers of fibrosis (Table S3) were included in this study and they were studied using 7 staining sets. We used 5 Cellomics Hitkits to track changes in cell proliferation (BrdU cell proliferation kit), apoptosis (Multiparameter apoptosis 1 kit and Caspase 3 activation kit), cell shape (Multiparameter apoptosis 1 kit), oxidative stress (Oxidative stress 1 kit) and cytokine activities (Smad3 and phospho-CREB activation kit). Five samples and their duplicates were separately stained using the 5 kits. The staining steps were carried out according to the manufacturer's instructions (Thermo Fisher Scientific, Rockford, Illinois) with the exception of the nuclear staining procedure. For all the staining protocols in this study, nuclei were separately stained (Hoechst 33258 diluted 1∶1000) after secondary antibody staining and incubated for 10 minutes under room temperature before the cells were washed and subjected to image acquisition.

In addition, two samples and their duplicates were separately stained with collagen type III antibody or double-stained with matrix metalloproteinase-2 (MMP-2) and tissue inhibitor of metalloproteinases-1 (TIMP-1) antibodies. LX-2 cells were fixed in pre-warmed 3.7% paraformaldehyde (Sigma-Aldrich, St Louis, MO, USA) in 37°C for 10 minutes and permeabilized with 1% Triton X-100 (Thermo Fisher Scientific, Rockford, Illinois) at room temperature for another 10 minutes before blocking with 10% BSA (Sigma, Canada). After 30 minutes blocking, the cells were incubated with either anti-collagen III antibody (diluted 1∶100, Santa Cruz Biotechnology) or a mixture of the MMP-2 and TIMP-1 antibodies (anti-MMP-2 antibody was diluted 1∶1000, Santa Cruz Biotechnology; anti-TIMP-1 antibody was diluted 1∶100, Santa Cruz Biotechnology) for 2 hours at room temperature. After washing, the cells were incubated with fluorescein-conjugated affinity purified anti-rabbit IgG (H&L) (goat) (diluted 1∶200, Rockland, USA) or Texas red conjugated affinity purified anti-mouse IgG (H&L) (donkey) (diluted 1∶200, Rockland, USA) at room temperature for 1 hour, protected from light. Hoechst 33258 (diluted 1∶1000) was subsequently added for 10 minutes before the cells were washed and subjected to image acquisition.

### Image acquisition

Images were acquired using Cellomics ArrayScan VTI (Thermo Scientific) controlled by vHCS*™* Scan software version 6.1.4 (Build 6133). All images were taken with a LD Plan_Neofluar 20× air objective. 16 high-resolution images (1024×1024 pixels) were taken per well, which captured about 1000 to 2000 cells per experimental condition.

### Image processing and statistical analysis

There are about 100 cells captured per image. Image segmentation and feature extraction were performed with a modified evolving generalized Voronoi diagrams algorithm [20], in which individual cells were identified and 25 or 16 cytological features were extracted per cell, for samples with 3-channel or 2-channel staining respectively. These features described cellular shape, protein distribution and content. A complete list of cytological features is shown in Table S2. The efficacy predictor (E*_predict_*) was computed using Matlab R2009a with image processing and statistical toolboxes (material S1). In short, the raw data from *in vitro* experiments consists of multiple dimensions that include multiple cells in each treatment condition, drug concentrations, cellular features, and fibrotic markers. We reduced the data complexity in a 3-step statistical process (described in the material S1) and derived a *SAUC* value per fibrotic marker per drug. Subsequently, the E*_predict_* score was computed by a linear combination of the *SAUC* values. The optimized weight for each fibrotic marker in the linear combination was calculated and validated independently with the training and validation *in vivo* data sets respectively (described in the result section 4).

### Automation

**Figure 1:**
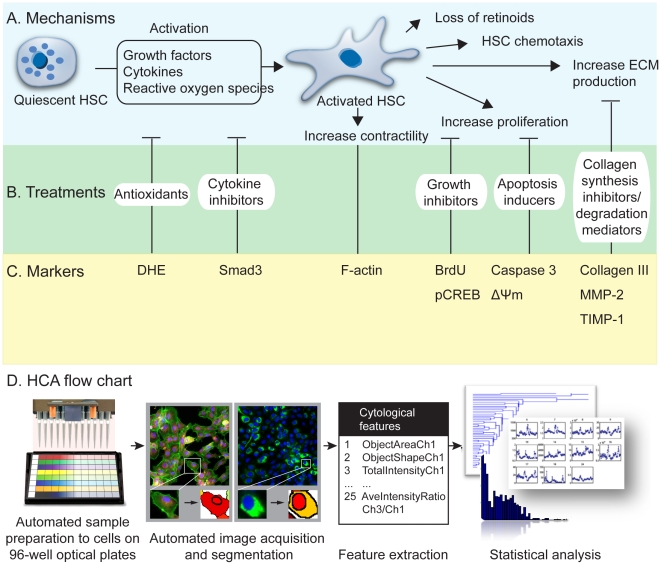
Principle for evaluation of anti-fibrotic drug efficacy. (A) Phenotypic changes of hepatic stellate cells during activation. (B) Potential sites for therapeutic interventions and (C) markers that track the effects of the interventions. (D) The high-content analysis system with 4 core components (sample preparation, automated image acquisition, image processing and statistical analysis).

During activation, HSCs undergo phenotypic changes such as increasing proliferation and ECM production (Fig. 1A) [21]. Many of these changes are potential therapeutic targets. We followed 5 such changes (Fig. 1B) with 10 chosen markers (Fig. 1C) using an HCA system that can be divided into 4 components: sample preparation, automated image acquisition, image processing and statistical analysis (Fig. 1D). All the sample preparation procedures including cell culture, drug preparation, drug treatment, cell viability assay and immunofluorescence staining were automated using a JANUS™ automated liquid handling system (Perkin Elmer).

## Results

We have developed an HCA-based quantitative assessment screen that uses the E*_predict_* value to correlate *in vitro* and *in vivo* anti-fibrotic drug responses. Subsequently the E*_predict_* value was used in two applications: predicting *in vivo* drug efficacy from *in vitro* data, and determining the cellular pathways that are common among the more effective anti-fibrotic drugs.

### All 10 markers of fibrosis captured drug-induced changes in LX-2 cells

In HCA, cells were treated with the 49 drugs at 11 concentrations, stained for 10 markers of fibrosis (Table S3), and imaged using automated microscopy. Cellular features such as the extent of changes in shape and marker intensity were then quantified for assessing the anti-fibrotic efficacies of the drugs.

**Figure 2:**
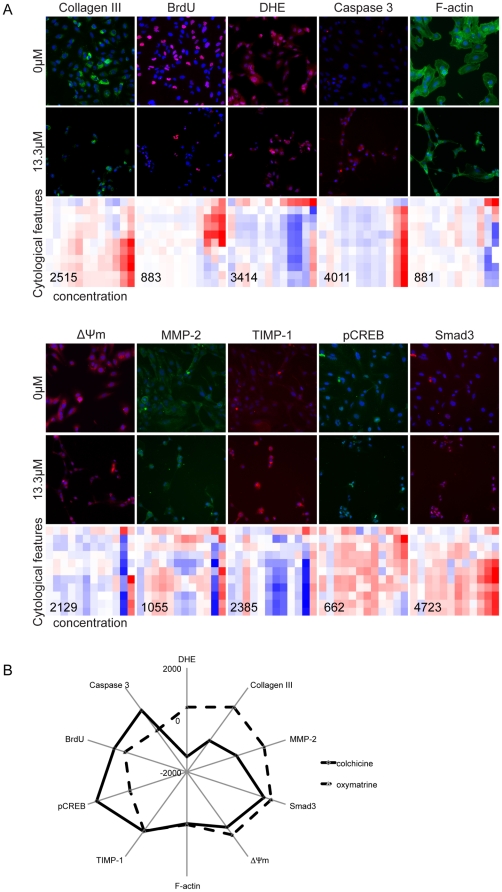
Images and quantification of the changes of LX-2 with drug treatment. (A) The cells are treated with or without 13.3 µM glycyrrhizin as indicated for 48 hours. Nuclei are stained (blue) in all the images; while 10 fibrotic markers are represented with either red or green colors. Heatmaps show the variations of the *KR* values for each of the cytological features (y-axis) with increasing drug concentrations from 0 µM to 13.3 µM (x-axis). Cytological features with similar variations are clustered together in the heatmaps. Drug-induced concentration-dependent changes can be clearly detected in the graphs. Numbers in the heatmaps are the *SAUC* values. (B) The *SAUC* values for drugs colchicine and oxymatrine.

Drug-induced changes can be clearly detected in the datasets; for example, glycyrrhizin caused an increase in apoptosis (*i.e*., increase in the caspase 3 level and decrease in the mitochondrial membrane potential ΔΨm, measured by Mitotracker Red) and a decrease in four other markers: proliferation (*i.e.*, bromodeoxyuridine (BrdU) positive cells), oxidative stress (*i.e.*, dihydroethidium (DHE) intensity), collagen (*i.e.*, collagen type III intensity), and TIMP-1 (*i.e.*, TIMP-1 intensity). The Smad3 marker for TGF-*β*1/fibrosis signaling was also studied. The ratio between nuclear and cytoplasmic intensities for Smad3 decreased with drug treatment, demonstrating reduced nuclear translocation and reduced activation of the protein. This suggests that glycyrrihizin can down-regulate the TGF-*β*1 signaling pathway. Furthermore, the total Smad3 level increased in cells treated with anti-fibrotic drugs; previous work showed that Smad3 is required for inhibiting HSC proliferation [22] (images in Fig. 2A).

### Changes in fibrotic markers *in vitro* are consistent with *in vivo* drug response

We used a modified evolving generalized Voronoi diagrams algorithm to identify individual cells from the images. 5 nuclear features and 11 cytoplasmic features per marker were extracted from each cell. These features quantitatively described cellular characteristics such as cell shape, protein expression levels and protein localization in the nucleus and cytoplasm (Table S2).

The cellular features from cells treated with various drug concentrations were normalized and combined to create a single *SAUC* score per fibrotic marker per drug (material S1). The *SAUCs* vary positively with the anti-fibrotic effects of a drug on the 10 markers. Briefly, we converted the cellular feature values into a Kolmogorov-Smirnov score [23] or ratio depending on whether a feature value has a unimodal or bimodal distribution. Both Kolmogorov-Smirnov score and ratio vary from -1 to 1 and the combined result was termed the *KR* value (Fig. 2A). A negative *KR* value represents a decreasing feature value (*e.g.* intensity) compared with the control; while a positive one represents increasing feature value. The *KR* values exhibit drug concentration-dependent changes shown by the color intensities in the heatmaps. The extent of changes of cells stained with a particular marker is then computed from the *KR* values and termed the *SAUC* score, which is the sum of the sign corrected area under the curve from a plot of *KR* values versus drug concentrations. The sign of the *SAUC* value was corrected to increase if the drug exhibits anti-fibrotic effects (material S1).

Each drug has 10 *SAUC* values corresponding to the 10 markers of fibrosis. *In vitro* drug effects can be assessed based on these values, and the results could be correlated to *in vivo* response. For example, oxymatrine exhibited a higher efficacy than colchine, as oxymatrine treated rats had lower histopathological scores, smaller collagen area in the liver tissue, and lower concentrations of the serum markers such as hyaluronic acid and procollagen III compared with colchicine treated rats [24]. From our HCA results, the *SAUC* values for at least half of the markers showed a higher value for oxymatrine than colchicine (Fig. 2B). In order to have a more quantitative comparison of the drug efficacies, our goal is to consolidate the 10 *SAUC* values into a single index as a drug efficacy predictor that is positively correlated with an *in vivo* drug efficacy index.

### An *in vivo* anti-fibrotic drug efficacy index ranks drugs based on their *in vivo* effects

Different weights will be assigned to the *SAUC* values to reflect the relative importance of each of the markers towards the overall efficacy. The weights should be chosen so that the overall index can reflect the *in vivo* response of a drug. Before we can do that, we need a numerical measure of the *in vivo* drug efficacy. Previous work that involved multiple drugs in a single *in vivo* study carried out the drug efficacy comparison by assessing the extent of fibrosis in liver biopsy samples, as well as the level of surrogate serum markers for liver fibrosis such as alanine aminotransferase (ALT) and aspartate aminotransferase (AST). Such an approach does not summarize the experimental results into a drug efficacy index for direct comparison and ranking of drugs within a single *in vivo* study or between studies. Here we analyzed the literature and an *in vivo* drug efficacy scoring system was computed based on histological scores.

Most of the *in vivo* studies reported in the literature were carried out in rat models. Although numerous such papers are available, there is no standard method to compare these results. To compare the *in vivo* drug efficacies, we have established an *in vivo* index based on pathologist-graded histological scores, which are considered a gold standard for quantifying the extent of fibrosis. A systematic search was performed on the reported *in vivo* effects of all 49 drugs on hepatofibrotic rats. The search yielded 28 papers from 1986 to 2009 with pathologist-graded histological scores, using CCl_4_, TAA, DMN, cisplatin, pig serum, high calorie diet or bile duct ligation induced fibrotic rats (Table S4). These studies can be further divided into preventive or treatment models, depending on whether a drug is given since the first injection of hepatotoxin or after liver fibrosis has been established.

To define a formula for *in vivo* drug efficacy, we attempted to combine the histological score of fibrotic animals without drug treatment (*S_c_*) and the histological score of drug treated animals (*S_t_*). The *in vivo* efficacy of a drug is expected to be positively correlated with the changes in histological scores between the control and drug-treated biopsy samples (*S_c_* - *S_t_*). In addition, the drug efficacy may also be positively dependent on the fibrosis severity, as there are observations that individuals with more advanced fibrosis are less likely to respond to treatment, hence these patients require drugs with higher efficacy [15]. A quantitative *in vivo* efficacy index (E*_in vivo_*) was computed as shown below:
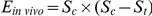
Both *S_c_* and *S_t_* were linearly converted to a 0–4 scale, which is a commonly used range for histological scores in several fibrosis scoring systems such as Metavir, Knodell and Ludwig [25]. If histological scores of a drug from multiple studies were available, the highest E*_in vivo_* value was chosen.

The severity of fibrosis induced by different hepatotoxins varies (*e.g.* E*_in vivo_* for silymarin is 0.8 for DMN treatment model, 3.1 and 6 for CCl_4_ treatment and preventive models); hence the indices are only comparable within the same fibrosis model. Subsequent correlation analysis was conducted using studies with long-term (>3 week) drug treatment, and fibrotic models with at least 3 drugs. The *in vivo* results satisfying these criteria are summarized in Table 1. CCl_4_ preventive and treatment models have 5 drugs in common; we found that three of these drugs: silymarin, malotilate and pioglitazone have the same relative ranking in both models while PCN and taurine didn't follow the ranking. Interestingly subsequent analysis showed that both PCN and taurine were outliners in the *in vitro*-*in vivo* correlation plots.

**Table 1:** Indexing of anti-fibrotic drugs from *in vivo* data.

Animal models	Drugs	histological score (DMN alone) (Sc)	histological score (with drug) (St)	E*_in vivo_*:(Sc-St)xSc
**DMN induced fibrosis (treatment)**	silymarin [40]	2	1.6	0.8
	thalidomide [41]	1.56	0.89	1
	tetrandrine [40]	2	1.3	1.4
	colchicine [42]	3.8	2.3	5.7
**CCl_4_ induced fibrosis (treatment)**	silymarin [43]	3.4	2.5	3.1
	5-Pregnen-3β-ol-20-one-16α-carbonitrile (PCN) [44]	3.84	2.8	4
	malotilate [45]	3.76	2.67	4.1
	rosmarinic acid [43]	3.4	2.1	4.4
	pioglitazone [46]	4	2.63	5.5
	taurine [47]	3.33	1.33	6.7
**CCl_4_ induced fibrosis (preventive)**	PCN [44]	3.6	3.68	−0.3
	taurine [48]	3.03	1.87	3.5
	melatonin [49]	3.38	2.25	3.8
	oxymatrine [50]	3.76	2.43	5
	silymarin [51]	4	2.5	6
	malotilate [45]	2.91	0.76	6.3
	EGCG [52]	3.58	1.5	7.4
	pioglitazone [46]	4	1.94	8.2

All data are taken from the literature using dimethylnitrosamine (DMN) treatment, carbon tetrachloride (CCl_4_) treatment, or CCl_4_ preventive fibrotic rat models. Histological scores are linearly converted to a scale from 0 to 4. E*_in vivo_* is established as shown. Drugs under each animal model are sorted according to increasing E*_in vivo_*. Silymarin, malotilate and pioglitazone have the same relative ranking in CCl_4_ treatment and preventive models.

The calculated E*_in vivo_* is an attempt to capture the therapeutic efficacy of drugs on human patients. There are relatively few studies suitable for directly comparing drug effects on human patients due to variations in experimental design. In one example, two similar clinical studies using colchicine and silymarin on patients with cirrhosis due to any primary insults showed that colchicine led to 75% 5-year survival rate [26], while silymarin led to 58% 4-year survival rate [27]. E*_in vivo_* agrees with these reports that colchicine has a higher value (5.7) than silymarin (0.8) (Table 1).

### An *in vitro* efficacy predictor E*_predict_* that positively correlates with the E*_in vivo_* value of a drug

The *SAUC* values for the majority of drugs showed a weak positive correlation with the E*_in vivo_* (Fig. S1: ΔΨm, TIMP-1, DHE, pCREB and Smad3). We investigated if we could further enhance this correlation by applying weights (0, 1 or 2) to the *SAUC* values. 0 indicates no contribution of the marker to the positive correlation; while 2 indicates strong contribution of the marker to the positive correlation. The E*_in vivo_* values from the CCl_4_ treatment model were used as the training dataset to find the optimized weights.

**Figure 3:**
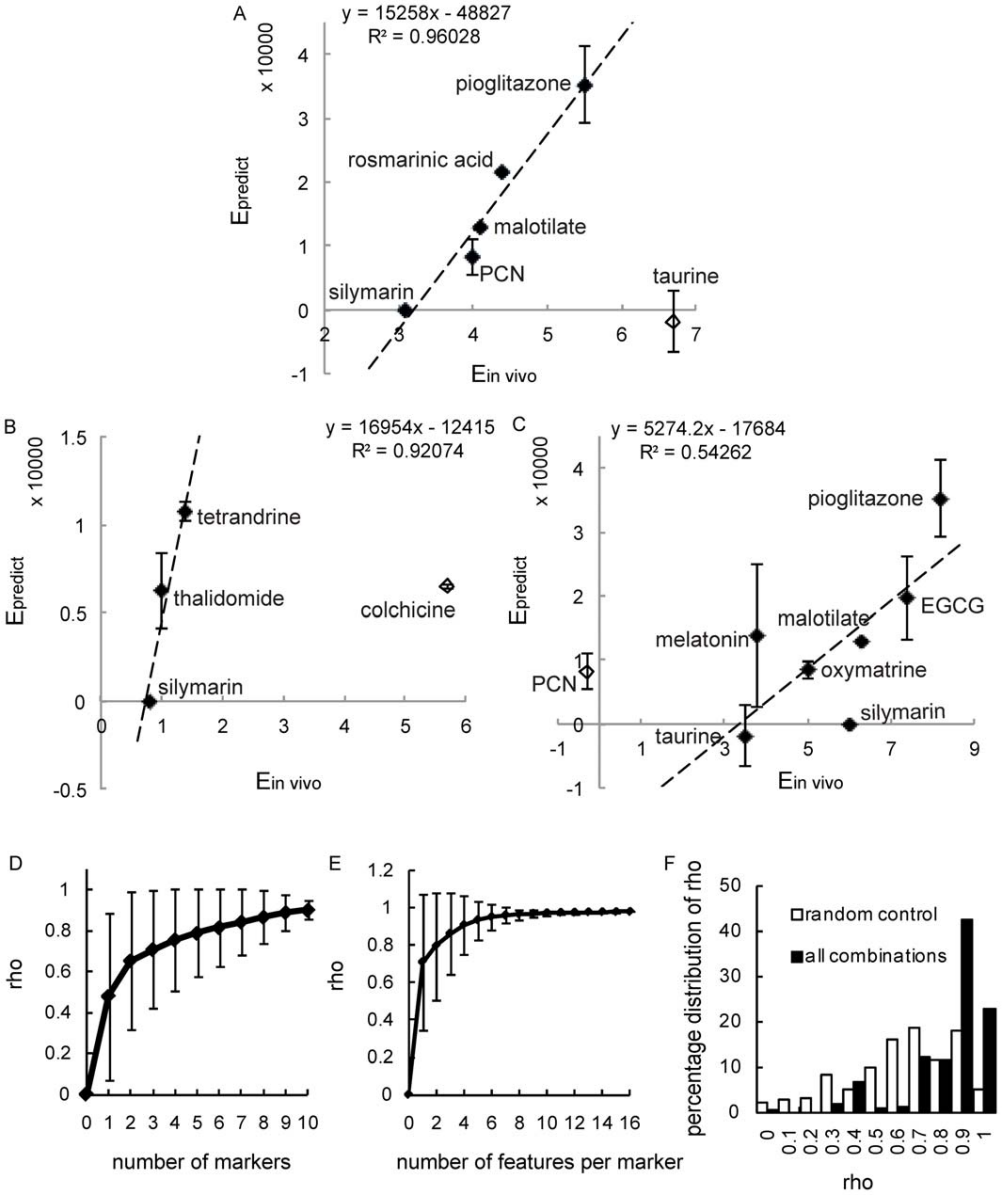
Correlation between E*_predict_* and E*_in vivo_*. (A) Optimization of E*_predict_*. E*_predict_* is computed as a weighted combination of the features with weights optimized using Spearman's rank correlation test to best correlate with E*_in vivo_* from the CCl_4_ treatment model. (B, C) Blind validations of *in vitro*-*in vivo* correlation between E*_predict_* and E*_in vivo_* from two independent datasets containing DMN treatment and CCl_4_ preventive models respectively. The linear relationship is highlighted using linear regression lines in all (A, B and C). The equations of the linear regression lines and the R^2^ values are computed without considering the outliers in the graphs (*i.e.*, taurine in A, colchicine in B and PCN in C). (D) The relationship between the average *rho* value and the number of markers. (E) The relationship between average *rho* and the number of features per marker. Error bars represent standard deviation. (F) The percentage distribution of *rho* is plotted for all possible combinations of the 3 weights and 10 markers. The random control was done by randomizing the relative ranks of the *in vivo* drug efficacies for the Spearman's rank correlation test.

All possible linear combinations of the 3 weights with 10 markers (3^10^ combinations) were subjected to the Spearman's rank correlation test [28] against E*_in vivo_* from CCl_4_ fibrosis model. One outlier was allowed in the analysis, as the sample size is relatively small. The Spearman's rank correlation coefficient *rho* ranges from 0 to 1, where 1 means perfect rank correlation (excluding the outlier), and 0 means the opposite order. The optimized weight for each marker was determined to be the value with the highest frequency occurrence out of all cases which achieved *rho* = 1 (Fig. S2). High weight implies high importance of the marker towards a strongly positive correlation. The optimized weights yielded the following efficacy predictor (E*_predict_*), computed as the linear combination of the 10 optimized weights with the *SAUC* values:
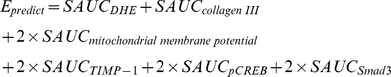
A greater E*_predict_* represents a higher drug efficacy and all negative values were assigned to 0 as no efficacy. We also incorporated an additional step to identify drugs with non-specific effects that cause an increase in collagen expression (material S2 and Fig. S3); their E*_predict_* values were also assigned to 0 (Table 2). Fig. 3A shows that the E*_predict_* values had a good correlation with the E*_in vivo_* from the CCl_4_ treatment model, which was used for optimizing the weights. Although the statistical approach used was to optimize the ranking order of the drugs, a linear relationship was observed in the plot. Taurine was found to be an outlier. Its relatively high *in vivo* efficacy compared with the other drugs used in CCl_4_ treatment model in Table 1 might be due to the much higher drug concentration used in the study (1200 mg/kg daily) compared with a typical drug concentration (<100 mg/kg daily) for the rest of the drugs.

**Table 2:** List of E*_predict_* values for all the drugs.

Drugs	E*_predict_*
taxifolin	0
taurine	0
curcumin	0
resveratrol	0
silymarin	0
minoxidil sulphate	0
simvastatin	0
genistein	0
lovastatin	0
PTK787/ZK22258 (PTK/ZK)	0
Y27632	0
rotenone	0
AG1295	0
paclitaxel	0
aphidicolin	0
nocodazole	0
pentoxifylline	5175
matrine	5295
astragaloside IV	5496
thalidomide	6263
colchicine	6487
TGF*β* inhibitor V	6974
gliotoxin	7086
5-Pregnen-3β-ol-20-one-16α-carbonitrile (PCN)	8203
camostat mesylate	8231
imatinib mesylate	8454
oxymatrine	8528
pirfenidone	8837
minoxidil	9069
AG1296	9154
somatostatin	10057
MG132	10669
tetrandrine	10747
telmisartan	11467
malotilate	12941
melatonin	13728
fasudil HCl	14295
olmesartan medoxomil	15959
silybin	18138
TGF*β* inhibitor III	18315
tranilast	19594
epigallocatechin gallate (EGCG)	19704
bortezomib	21047
rosmarinic acid	21435
berberine chloride	21983
staurosporine	25015
glycyrrhizin	25728
pioglitazone	35226
sulfasalazine	39437

To validate that E*_predict_* is a robust anti-fibrotic drug efficacy predictor that can correlate with the *in vivo* data from other rodent fibrosis models different from the training dataset; we tested the ability of E*_predict_* to correlate with two “blind” *in vivo* datasets. We drew two additional correlation plots of E*_predict_* against E*_in vivo_* from DMN treatment (Fig. 3B) and CCl_4_ preventive models (Fig. 3C). E*_predict_* was kept the same as computed for the CCl_4_ treatment model. A positive correlation as well as a linear relationship between E*_predict_* and E*_in vivo_* was again observed in both plots. To further prove that this relationship does not depend on the choice of the training set of data, similar results were obtained if DMN treatment or CCl_4_ preventive models were used as the training dataset instead of the CCl_4_ treatment model (data not shown).

Fig. 3D and E demonstrate how *rho* varies with the number of markers and the number of cytological features, respectively. Both curves reach a plateau before or at our experimental configuration of 10 markers and 16 features per cell, showing that our study design is sufficient for the anti-fibrotic correlation study. We next test the robustness of the experimental configuration by shuffling the weights in the E*_predict_* formula; Fig. 3F shows the plot for the percentage distribution of *rho* for all possible combinations of the 3 weights and 10 markers. There is a 23% chance of *rho* being equal to 1, which is significantly higher than the random control (5% chance of *rho* being equal to 1) in which the relative ranks were randomized before applying the Spearman's rank correlation test. This demonstrates that a positive correlation between the *in vitro* and *in vivo* indices can be achieved even if the optimized set of weights is not used, implying that the weighting procedure of our system is not vulnerable to high background noise. The *in vitro SAUC*s have good predictive value alone, and the E*_predict_* weighting of the *SAUC*s optimizes their correlation and augments their predictive power.

### The *in vivo* histological scores can be estimated from E*_predict_*

The linear relationship observed in all the three correlation plots may be used to generate predictions of *in vivo* drug efficacies based on *in vitro* measurements. Since all the *in vivo* data from long-term drug treatment studies have been used either to build or validate the *in vitro*-*in vivo* correlation, we now turn to short-term drug treatment (<3-week treatment including single injection) as another source of information for validating the predictive capability of E*_predic_*_***t***_. One such study is available, concerning sulfasalazine. We would like to use *in vitro* E*_predic_*_***t***_ values generated from HCA to predict *in vivo* histological scores. Since E*_predic_*_***t***_ was optimized with data from long-term studies, the predicted histological scores should be similar to long-term drug treatment outcomes. The histological scores from short-term studies are expected to be slightly higher than our prediction, because prolonging the treatment with the same drug used in the short-term studies may further improve the fibrotic status and decrease the histological scores.

The E*_predic_*_***t***_ value of sulfasalazine is 39437; using the linear relationship from the CCl_4_ treatment model (equation in Fig. 3A), the E*_in vivo_* value is calculated to be 5.8. Assuming the histological score of rat livers with CCl_4_ induced fibrosis and no anti-fibrotic treatment is 3.0 (same as in [29]), a long-term treatment with sulfasalazine is predicted to reduce the fibrosis histological score to 1.1. A short-term study on rat CCl_4_ treatment model reported that a single injection of sulfasalazine reduced the fibrosis score to 1.5 compared with 3.0 in untreated CCl_4_-only livers [29]. The results agreed with our expectation, showing that the *in vivo* histological scores can be estimated from E*_predict_*.

### High-efficacy drugs tend to target proliferation, apoptosis and contractility of HSCs

All drugs were grouped into 3 categories based on their E*_predict_* values. The negative (n) group was defined to include all drugs with E*_predict_* equivalent to 0. Seven drugs with the highest E*_predict_* values were placed into the very positive (vp) group. The rest of the drugs were in the positive (p) group. Before proceeding to quantitative analysis, we firstly remark on some trends and background about the categorized drugs. The n group has 16 drugs including 6 anti-oxidants, two HMG-CoA reductase inhibitors, simvastatin and lovastatin, and all 4 non-specific control compounds not related to fibrosis. Tranilast from the p group showed anti-fibrotic effects in renal and liver fibrosis [30], [31], and it has a relatively high E*_predict_* value of 19594. It has been reported as a positive drug in another high-throughput screening study [8]. In the vp group, glycyrrhizin, a herbal extract from licorice, showed positive effects on patients with hepatitis C [32]. Pioglitazone is another highly effective drug in the vp group that has been subjected to multiple advanced stage clinical studies [14]. It is one of the peroxisomal proliferator activated receptor gamma ligands, which have overall higher efficacies on human patients than colchicine, interferon gamma, and angiotensin receptor blockers [15].

**Figure 4:**
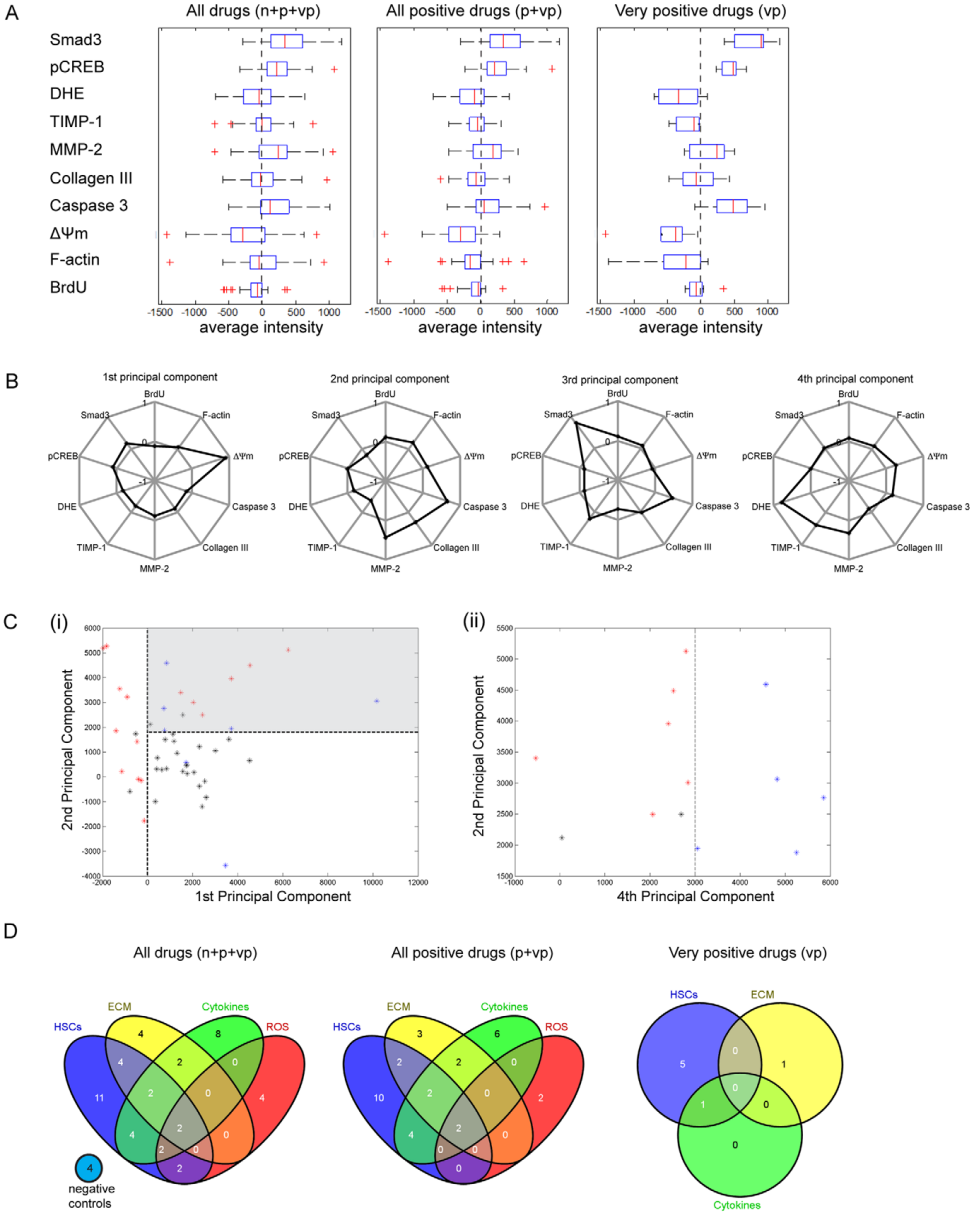
Distinctive characteristics of the negative (n), positive (p), and very positive (vp) groups of drugs. (A) The average intensities of the 10 makers for all drugs (n+p+vp), all positive (p+vp) drugs and the vp group of drugs are shown in the boxplots, where inter-quartile ranges are represented by boxes. Whiskers represent 1.5 times the inter-quartile range and any data (outliers) beyond the whiskers are shown using red +. (B) Principal component analysis (PCA) is done using data from the vp group. The top 4 principal components explain 95% of the cumulative variance in the system. (Ci) All 49 drugs are mapped to the first and second principal component coordinates. Drugs in the gray box in (Ci) are mapped to the second and fourth principal component coordinates in (Cii). The vp group (blue) is found to have relatively large values in the first, second and fourth principal components; while the p group (black) has positive values in the first principal component, but relatively low values in the second principal component. (D) All drugs (n+p+vp), all positive (p+vp) drugs and the vp group of drugs are classified into 4 categories according to their mechanisms of action.

The mean *KR* values of the average intensity for fibrosis markers were represented as boxplots for the n, p and vp groups of drugs (Fig. 4A). Fewer outliers (red plus) were observed in the plot for the vp group compared with that for all the drugs (n+p+vp), showing that drugs with high efficacies have similar cellular effects and probably have similar cellular targets.

A principal component analysis (PCA) was carried to detect the set of markers that carry the most information, which could reflect the importance of the underlying pathways. We found that the top 4 principal components built from *SAUC* values from drugs in the vp group explained more than 95% of the cumulative variance in the system. The first principal component mainly captures variation in ΔΨm, which plays an important role in the apoptotic pathway. The second principal component mainly captures variations in caspase 3 (also apoptosis), collagen III (ECM), MMP-2 (ECM) and TIMP-1 (ECM) (Fig. 4B). The three groups of drugs with different levels of efficacy can be well separated when mapped to the first, second and fourth principal component coordinates (Fig. 4C). The vp group (blue) is found to have relatively large values in the first, second and fourth principal components; while the p group (black) has positive values in the first principal component, but relatively low values in the second principal component. These results showed that apoptosis is an attractive anti-fibrotic target, while targeting ECM directly is also effective. Interestingly ΔΨm and caspase 3 did not co-vary with each other in the first and second principal components, suggesting that highly effective anti-fibrotic drugs target distinct sub-pathways of apoptosis: either the intrinsic mitochondria-dependent pathway, or caspase 3-dependent non-mitochondrial pathways. As a result, multiple apoptotic markers are needed to measure the effect of an anti-fibrotic drug on HSC apoptosis. In addition, MMP-2 and TIMP-1 have expected roles in the PCA analysis, being somewhat important, and often co-varying inversely with each other.

To validate the finding that apoptosis is an attractive anti-fibrotic target, the primary mechanism of action of each drug was found from the literature (Table S5) and was broadly categorized into 4 targets [33]. The target “cytokine” includes drugs targeting cytokines such as TGF-*β*1 and PDGF activities; the target “ECM” includes drugs inhibiting collagen synthesis or promoting degradation; the target “ROS” includes all anti-oxidants; and the target “HSCs” includes all other aspects including drugs targeting HSC proliferation, apoptosis or contractility. Drugs were allowed to be in 1 or multiple categories to account for the multiple signaling pathways a drug may be involved in; however, secondary mechanism of action (*e.g.* HCS apoptosis due to the anti-oxidative activity of a drug) is not included. The results were summarized in 4-way Venn diagrams (Fig. 4D). The 49 drugs showed a balanced distribution in each of the 4 categories. However, the more effective drugs seem to have their primary effects on HSCs directly, which agrees with the PCA result that the HSC apoptosis pathway is a potent drug target.

## Discussion

Suppressing collagen production or reducing HSC viability represents an important anti-fibrotic drug screening strategy. Single-parameter *in vitro* studies have relatively poor correlation with *in vivo* drug efficacy; while multi-parameter *in vitro* studies are easy to perform but difficult to interpret. In this paper, we take a systematic statistical approach to the problem of correlating multi-parameter HCA screening against published *in vivo* drug effects. Our HCA system includes 10 fibrotic markers and 16 imaging features per marker, which follow changes in reactive oxygen species, TGF-*β*, proliferation, apoptosis, collagen regulation and cell contractility. Using a limited subset of the *in vivo* literature, we compute an optimized interpretation of the *in vitro* data, called E*_predict_*, to predict *in vivo* drug efficacy. Then we test the performance of the E*_predict_* values on two different subsets of the *in vivo* literature. We find that E*_predict_* is able to identify drugs with anti-fibrotic effects, and also be able to distinguish drugs with moderate and high efficacies.

Studies of *in vitro*-*in vivo* comparative efficacy can help select promising categories of drugs to be given priority in the drug discovery pipeline. However, it is challenging to perform such analysis from limited *in vivo* literature. The preclinical and clinical results of many drugs are often lacking, incomplete or inconclusive. Even when *in vivo* data is available, the histological scores may not be assessed, while other serum markers such as ALT and AST may not directly reflect fibrosis severity [34]. Another important concern is the inter-observer variability by pathologists doing histological examination of biopsy samples [35]. This intrinsic baseline error seems to be well tolerated and low enough not to mask the linear relationship between data from *in vitro* cell culture and *in vivo* rat models in this study.

In this study the E*_predict_* value was derived from HCA and a limited training set of *in vivo* data, but its magnitude showed strong positive correlation with most of the available *in vivo* scores from fibrotic rat models, including blinded data sets that were reserved for validation purposes. The level of the *in vitro* efficacy was assessed by the overall effect of a drug on multiple pathways and partially reflects the complex *in vivo* response. It is interesting to see that a linear relationship with R^2^>0.9 exists between the *in vitro* and *in vivo* data for CCl_4_ and DMN fibrotic treatment models; while a weaker linear correlation (R^2^ = 0.54) was observed for the CCl_4_ preventive model. In the latter, fibrosis causing agents such as CCl_4_ and drugs were given together to rats. As a result, many of the drugs showing positive effects are protecting hepatocytes from toxins or preventing HSC activation, rather than inducing fibrosis regression. Since an activated HSC cell line is used in our screening platform, it is more closely mimicking the treatment model; hence a stronger linear relationship exists for both CCl_4_ and DMN treatment models. Furthermore, anti-oxidants work by preventing HSC activation induced by free radicals. This group of drugs can be considered preventive drugs, more than treatment drugs, which agrees with our result that most of the anti-oxidants have lower E*_predict_* values.

The ability of cell culture models to predict *in vivo* drug effects is limited by many fundamental constraints. For example, drugs might be able to improve liver fibrosis by improving vascular flow or liver architecture, such as the angiotensin II receptor antagonists losartan and candesartan. Some drugs are metabolized by hepatocytes into secondary compounds with different effects; and such effects cannot be foreseen *in vitro* using HSC monocultures. This study investigated the effects of drugs on HSCs only. Our system is not suitable to substitute for animal trials, but we recommend it for prioritizing the selection of drugs to enter animal trials.

Interestingly, we have observed promising pieces of evidence that the E*_predict_* score can potentially be correlated with data from human clinical trials. For example, the group of drugs with relatively high E*_predict_* scores (e.g. pioglitazone [36] and glycyrrhizin [37]) gave more promising results in human clinical trials than the group of drugs with low E*_predict_* scores (e.g. colchicine [38] and silymarin [39]). Furthermore, drugs with lower E*_predict_* scores generally have fewer *in vivo* publications than drugs with higher E*_predict_* scores. Such relationship may be partially due to the fact that the hepatic stellate cell line used in this study is from human origin.

In conclusion, our anti-fibrotic drug screening platform is able to index and rank drugs according to their *in vitro* efficacy. The *in vitro* index system positively correlates with the *in vivo* histological scores, which shows that our *in vitro* cell-based system has some predictability of the *in vivo* drug response. Furthermore, drugs with higher efficacies are found to exert their effects through directly modulating HSC proliferation, apoptosis or contractility.

## Supporting Information

Figure S1 **Correlation between** ***SAUC*** **and E** ***_in vivo_*** **for rat CCl4 treatment model.**
(TIF)

Figure S2 **Pie charts showing the chance of occurrence of weights in all cases where the Spearman's rank correlation coefficient** ***rho*** **achieves 1 for the training set of data.** The optimized weight for each marker is the value with the highest occurrence indicated with a * in each pie chart, which implies the relatively higher importance of the marker towards contributing to a stronger positive correlation.
(TIF)

Figure S3 **Images and quantification of hepatic stellate cells LX-2 with collagen III immuno-fluorescence staining.** Cells are treated with (A) pioglitazone, (B) EGCG, or (C) aphidicolin at the indicated concentrations for 48 hours (blue: nuclei; green: collagen III). The amount of collagen III in the cytoplasmic region is quantified and represented as the percentage of total collagen III intensity with respect to the control without drug treatment. Error bars represent standard deviation from 2 replicate datasets.
(TIF)

Table S1 **List of drugs and their highest working concentrations.**
(DOC)

Table S2 **List of cellular features according to staining sets.** 10 fibrotic markers were studied using 7 staining sets. S1: Cellomics BrdU cell proliferation kit (BrdU). S2: Cellomics multiparameter apoptosis 1 kits (F-actin, mitochondrial membrane potential, ΔΨm). S3: Cellomics caspase 3 activation kit (caspase 3). S4: Immunofluorescence staining of collagen III (collagen III). S5: Immunofluorescence staining of MMP-2 and TIMP-1 (MMP-2, TIMP-1). S6: Cellomics oxidative stress 1 kit (DHE). S7: Cellomics Smad3 and phospho CREB activation kit (Smad3, pCREB). Ch1: channel 1 for nuclear staining (blue). Ch2: channel 2 for protein staining (red or green for two-channel images; green for three-channel images). Ch3: channel 3 for protein staining (red for three-channel images). The nuclear region is defined by the Ch1 object mask. The cytoplasmic region that is positive for protein staining is defined by Ch2 (or Ch3) object mask. Nuclei were stained in all 7 staining sets. Since nuclear features (features 1 to 5) are similar regardless of the protein stainings in channel 2 and 3, they are only considered once in S1. S1, S3, S4 and S6 were duble-stained with one nuclear dye (Ch1) and one dye for a marker protein (Ch2). They do not have features related to Ch3.
(DOC)

Table S3 **List of references for the 10 markers of fibrosis.**
(DOC)

Table S4 **List of papers with pathologist-graded histological scores on fibrotic rats from 1986 to 2009.**
(DOC)

Table S5 **Mechanisms of action of the drugs.** All 49 drugs are classified based on their mechanisms of action from the literature.
(DOC)

Material S1 **Computing E** ***_predict_*** **from cellular feature values.**
(DOC)

Material S2 **Method to identify drugs with non-specific effects from** ***in vitro*** **HCA analysis.**
(DOC)
