# Supplementary figures and images for: Predicting In Vivo Anti-Hepatofibrotic Drug Efficacy Based on In Vitro High-Content Analysis
Source: PLoS One. 2011 Nov 2;6(11):e26230. doi: 10.1371/journal.pone.0026230 (PMC3206809; doi:10.1371/journal.pone.0026230)

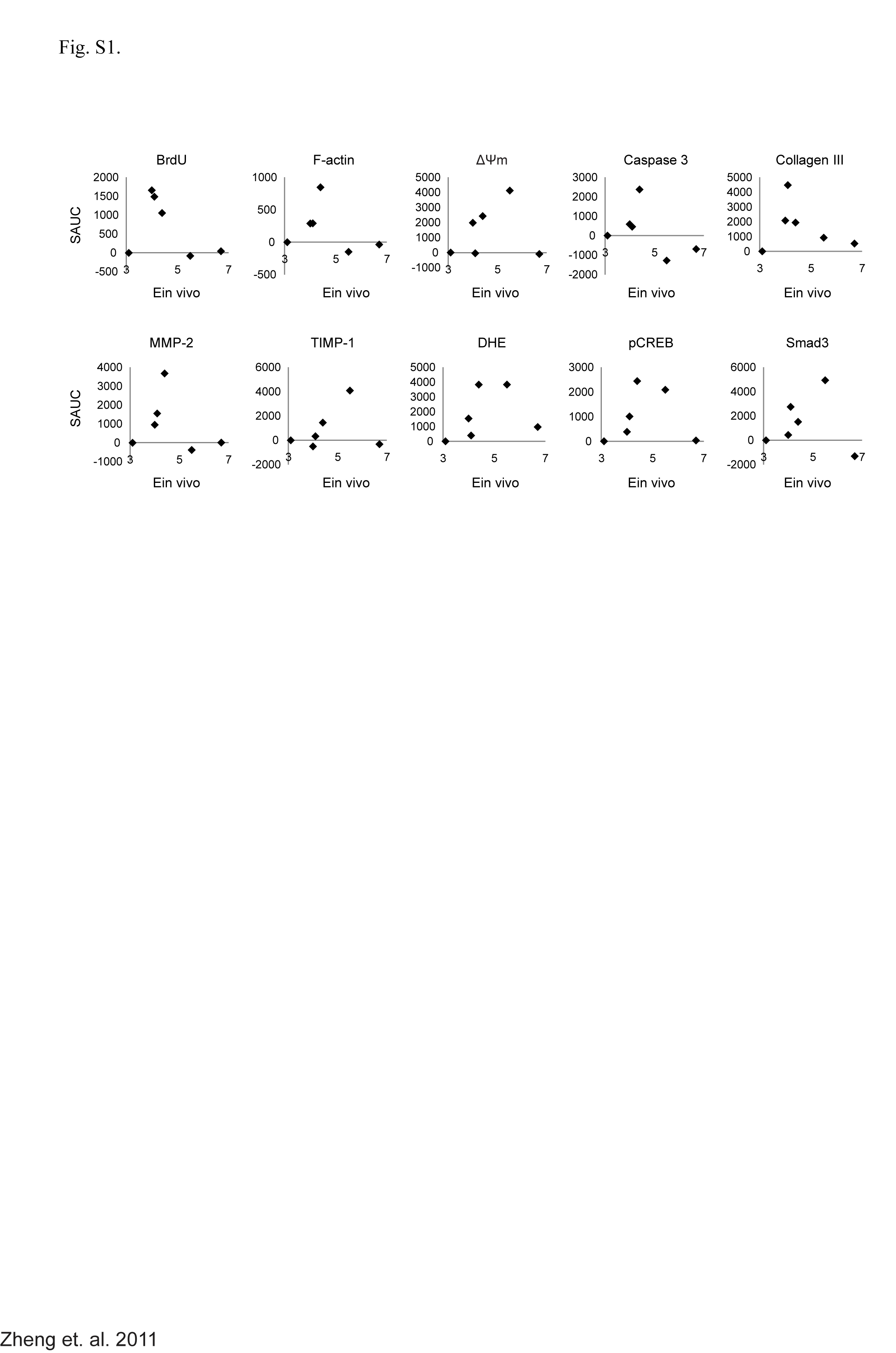

Supplement: Figure S1 — Correlation between SAUC and E in vivo for rat CCl4 treatment model. (TIF) [file pone.0026230.s001.tif]

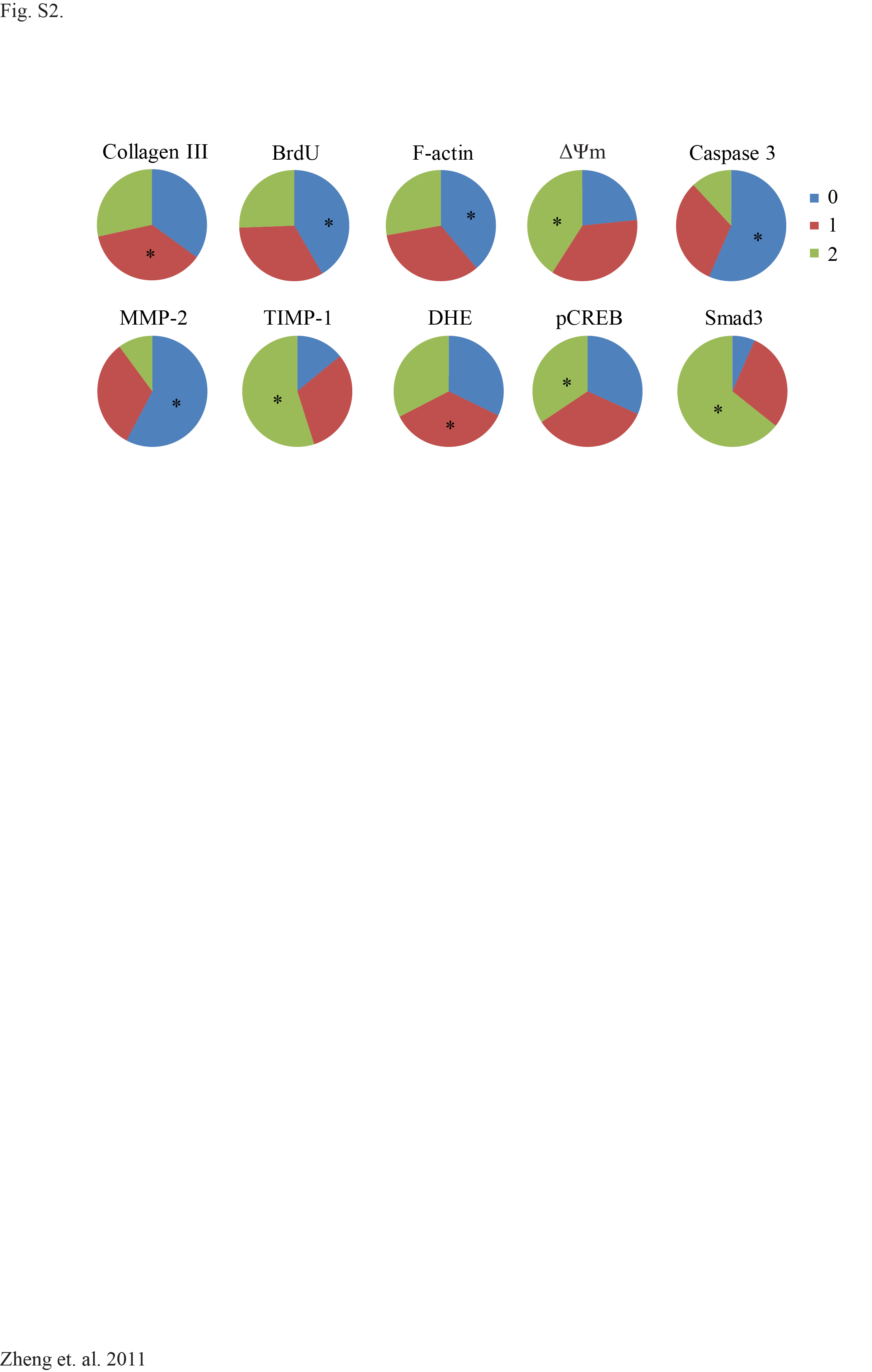

Supplement: Figure S2 — Pie charts showing the chance of occurrence of weights in all cases where the Spearman's rank correlation coefficient rho achieves 1 for the training set of data. The optimized weight for each marker is the value with the highest occurrence indicated with a * in each pie chart, which implies the relatively higher importance of the marker towards contributing to a stronger positive correlation. (TIF) [file pone.0026230.s002.tif]

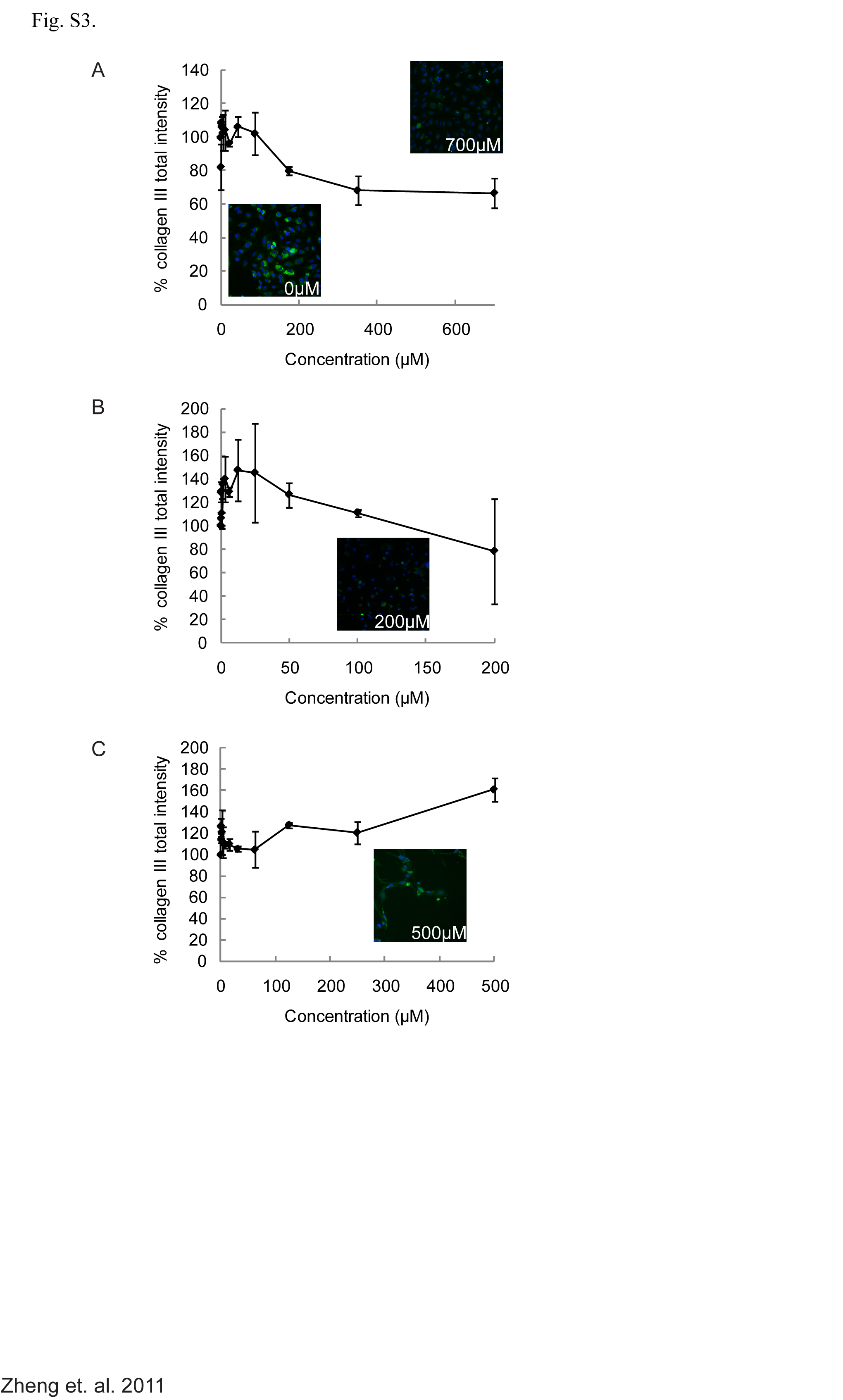

Supplement: Figure S3 — Images and quantification of hepatic stellate cells LX-2 with collagen III immuno-fluorescence staining. Cells are treated with (A) pioglitazone, (B) EGCG, or (C) aphidicolin at the indicated concentrations for 48 hours (blue: nuclei; green: collagen III). The amount of collagen III in the cytoplasmic region is quantified and represented as the percentage of total collagen III intensity with respect to the control without drug treatment. Error bars represent standard deviation from 2 replicate datasets. (TIF) [file pone.0026230.s003.tif]
