# Supplementary material for: Predicting In Vivo Anti-Hepatofibrotic Drug Efficacy Based on In Vitro High-Content Analysis
Source: PLoS One. 2011 Nov 2;6(11):e26230. doi: 10.1371/journal.pone.0026230 (PMC3206809; doi:10.1371/journal.pone.0026230)
Supplement: Table S1 — List of drugs and their highest working concentrations. (DOC) [file pone.0026230.s004.doc]

|  | **Drug name** | **Company** | **Cat. No** | **Highest working concentration (µM)** |
| --- | --- | --- | --- | --- |
| 1 | AG1296 | Merck (Calbiochem) | 658551 | 100 |
| 2 | curcumin | Merck (Calbiochem) | 239802 | 10 |
| 3 | epigallocatechin gallate (EGCG) | Merck (Calbiochem) | 324880 | 200 |
| 4 | resveratrol | Merck (Calbiochem) | 554325 | 250 |
| 5 | silymarin | Sigma | 254924 | 250 |
| 6 | taxifolin | Merck (Calbiochem) | 580553 | 125 |
| 7 | pentoxifylline | Merck (Calbiochem) | 516354 | 1000 |
| 8 | minoxidil sulphate | Merck (Calbiochem) | 475850 | 500 |
| 9 | colchicine | Merck (Calbiochem) | 234115 | 0.01 |
| 10 | TGF*β* inhibitor III | Merck (Calbiochem) | 616453 | 62.5 |
| 11 | TGF*β* inhibitor V | Merck (Calbiochem) | 616456 | 62.5 |
| 12 | AG1295 | Merck (Calbiochem) | 658550 | 100 |
| 13 | silybin | Sigma | S0417 | 500 |
| 14 | minoxidil | Sigma | M4145 | 250 |
| 15 | paclitaxel | Merck (Calbiochem) | 580555 | 0.016 |
| 16 | aphidicolin | Merck (Calbiochem) | 178273 | 500 |
| 17 | nocodazole | Merck (Calbiochem) | 487928 | 0.1 |
| 18 | staurosporine | Merck (Calbiochem) | 569397 | 100 |
| 19 | rotenone | Sigma | R8875 | 1 |
| 20 | genistein | Sigma | G6776 | 500 |
| 21 | bortezomib | Selleck chemicals | S1013 | 0.005 |
| 22 | imatinib mesylate | Selleck chemicals | S1026 | 15 |
| 23 | MG132 | Calbiochem | 474790 | 6.25 |
| 24 | gliotoxin | Sigma | G9893 | 0.15 |
| 25 | camostat mesylate | Tocris Bioscience | 3193 | 1000 |
| 26 | pirfenidone | Sigma | P2116 | 2000 |
| 27 | lovastatin | Tocris Bioscience | 1530 | 100 |
| 28 | PTK787/ZK22258 (PTK/ZK) | Selleck | S1101 | 250 |
| 29 | simvastatin | Sigma | S6169 | 50 |
| 30 | taurine | Sigma | T0625 | 4000 |
| 31 | Y27632 | Selleck | S1049 | 500 |
| 32 | thalidomide | Sigma | T144 | 5000 |
| 33 | 5-Pregnen-3β-ol-20-one-16α-carbonitrile (PCN) | Sigma | P0543 | 1000 |
| 34 | berberine chloride | Sigma | B3251 | 250 |
| 35 | tetrandrine | Sigma | 365629 | 6.25 |
| 36 | sulfasalazine | Sigma | S0883 | 5000 |
| 37 | olmesartan medoxomil | Toronto research chemicals inc. | O550000 | 308 |
| 38 | rosmarinic acid | Tocris Bioscience | 0630 | 700 |
| 39 | matrine | Sigma | M5319 | 5000 |
| 40 | fasudil HCl | Tocris Bioscience | 0541 | 62.5 |
| 41 | tranilast | Tocris Bioscience | 1098 | 1000 |
| 42 | melatonin | Sigma | M2675 | 1000 |
| 43 | glycyrrhizin | Merck (Calbiochem) | 356780 | 13.3 |
| 44 | somatostatin | Merck (Calbiochem) | 05-23-0850 | 10 |
| 45 | malotilate | Selleck | S1137 | 267 |
| 46 | oxymatrine | Wako reagents | 150-01511 | 3780 |
| 47 | astragaloside IV | Sigma | 74777 | 200 |
| 48 | telmisartan | Sigma | T8949 | 200 |
| 49 | pioglitazone | Toronto research chemicals inc. | P471000 | 700 |

Table S1. List of drugs and their highest working concentrations.
