# Supplementary material for: Predicting In Vivo Anti-Hepatofibrotic Drug Efficacy Based on In Vitro High-Content Analysis
Source: PLoS One. 2011 Nov 2;6(11):e26230. doi: 10.1371/journal.pone.0026230 (PMC3206809; doi:10.1371/journal.pone.0026230)
Supplement: Table S2 — List of cellular features according to staining sets. 10 fibrotic markers were studied using 7 staining sets. S1: Cellomics BrdU cell proliferation kit (BrdU). S2: Cellomics multiparameter apoptosis 1 kits (F-actin, mitochondrial membrane potential, ΔΨm). S3: Cellomics caspase 3 activation kit (caspase 3). S4: Immunofluorescence staining of collagen III (collagen III). S5: Immunofluorescence staining of MMP-2 and TIMP-1 (MMP-2, TIMP-1). S6: Cellomics oxidative stress 1 kit (DHE). S7: Cellomics Smad3 and phospho CREB activation kit (Smad3, pCREB). Ch1: channel 1 for nuclear staining (blue). Ch2: channel 2 for protein staining (red or green for two-channel images; green for three-channel images). Ch3: channel 3 for protein staining (red for three-channel images). The nuclear region is defined by the Ch1 object mask. The cytoplasmic region that is positive for protein staining is defined by Ch2 (or Ch3) object mask. Nuclei were stained in all 7 staining sets. Since nuclear features (features 1 to 5) are similar regardless of the protein stainings in channel 2 and 3, they are only considered once in S1. S1, S3, S4 and S6 were duble-stained with one nuclear dye (Ch1) and one dye for a marker protein (Ch2). They do not have features related to Ch3. (DOC) [file pone.0026230.s005.doc]

|  | **Features** | **Description** | **S1** | **S2** | **S3** | **S4** | **S5** | **S6** | **S7** |
| --- | --- | --- | --- | --- | --- | --- | --- | --- | --- |
| 1 | ObjectAreaCh1 | Area in pixels of Ch1 object | x |  |  |  |  |  |  |
| 2 | ObjectShapeCh1 | Circularity measured by the ratio of perimeter square to 4*pi*area of Ch1 object | x |  |  |  |  |  |  |
| 3 | TotalIntensityCh1 | Total intensity of all pixels in Ch1 object | x |  |  |  |  |  |  |
| 4 | AveIntensityCh1 | Average intensity of all pixels in Ch1 object | x |  |  |  |  |  |  |
| 5 | ObjectfragCh1 | Object fragmentation measured by standard deviation divided by mean of Ch1 object | x |  |  |  |  |  |  |
| 6 | ObjectAreaCh2 | Area in pixels of Ch2 object | x | x | x | x | x | x | x |
| 7 | ObjectShapeCh2 | Circularity measured by the ratio of perimeter square to 4*pi*area of Ch2 object | x | x | x | x | x | x | x |
| 8 | TotalIntensityCh2 | Total intensity of all pixels in Ch2 object | x | x | x | x | x | x | x |
| 9 | AveIntensityCh2 | Average intensity of all pixels in Ch2 object | x | x | x | x | x | x | x |
| 10 | ObjectfragCh2 | Object fragmentation measured by standard deviation divided by mean of Ch2 object | x | x | x | x | x | x | x |
| 11 | TotalIntensityCh3 | Total intensity of all pixels in Ch3 object |  | x |  |  | x |  | x |
| 12 | AveIntensityCh3 | Average intensity of all pixels in Ch3 object |  | x |  |  | x |  | x |
| 13 | ObjectfragCh3 | Object fragmentation measured by standard deviation divided by mean of Ch3 object |  | x |  |  | x |  | x |
| 14 | TotalIntensityNucCh2 | Total intensity of all pixels in Ch2 within Ch1 object mask | x | x | x | x | x | x | x |
| 15 | AveIntensityNucCh2 | Average intensity of all pixels in Ch2 within Ch1 object mask | x | x | x | x | x | x | x |
| 16 | TotalIntensityCytoCh2 | Total intensity of all pixels in Ch2 within Ch2 but not in Ch1 object mask | x | x | x | x | x | x | x |
| 17 | AveIntensityCytoCh2 | Average intensity of all pixels in Ch2 within Ch2 but not in Ch1 object mask | x | x | x | x | x | x | x |
| 18 | TotalIntensityCyto/NucCh2 | Ratio of TotalIntensityCytoCh2 to TotalIntensityNucCh2 | x | x | x | x | x | x | x |
| 19 | TotalIntensityNucCh3 | Total intensity of all pixels in Ch3 within Ch1 object mask |  | x |  |  | x |  | x |
| 20 | AveIntensityNucCh3 | Average intensity of all pixels in Ch3 within Ch1 object mask |  | x |  |  | x |  | x |
| 21 | TotalIntensityCytoCh3 | Total intensity of all pixels in Ch3 within Ch3 but not in Ch1 object mask |  | x |  |  | x |  | x |
| 22 | AveIntensityCytoCh3 | Average intensity of all pixels in Ch3 within Ch3 but not in Ch1 object mask |  | x |  |  | x |  | x |
| 23 | TotalIntensityCyto/NucCh3 | Ratio of TotalIntensityCytoCh3 to TotalIntensityNucCh3 |  | x |  |  | x |  | x |
| 24 | AveIntensityRatioCh2/Ch1 | Ratio of AveIntensityCh2 to AveIntensityCh1 | x | x | x | x | x | x | x |
| 25 | AveIntensityRatioCh3/Ch1 | Ratio of AveIntensityCh3 to AveIntensityCh1 |  | x |  |  | x |  | x |

Table S2. List of cellular features according to staining sets. 10 fibrotic markers were studied using 7 staining sets. S1: Cellomics BrdU cell proliferation kit (BrdU). S2: Cellomics multiparameter apoptosis 1 kits (F-actin, mitochondrial membrane potential, ΔΨm). S3: Cellomics caspase 3 activation kit (caspase 3). S4: Immunofluorescence staining of collagen III (collagen III). S5: Immunofluorescence staining of MMP-2 and TIMP-1 (MMP-2, TIMP-1). S6: Cellomics oxidative stress 1 kit (DHE). S7: Cellomics Smad3 and phospho CREB activation kit (Smad3, pCREB). Ch1: channel 1 for nuclear staining (blue). Ch2: channel 2 for protein staining (red or green for two-channel images; green for three-channel images). Ch3: channel 3 for protein staining (red for three-channel images). The nuclear region is defined by the Ch1 object mask. The cytoplasmic region that is positive for protein staining is defined by Ch2 (or Ch3) object mask. Nuclei were stained in all 7 staining sets. Since nuclear features (features 1 to 5) are similar regardless of the protein stainings in channel 2 and 3, they are only considered once in S1. S1, S3, S4 and S6 were duble-stained with one nuclear dye (Ch1) and one dye for a marker protein (Ch2). They do not have features related to Ch3.
