# Supplementary material for: Predicting In Vivo Anti-Hepatofibrotic Drug Efficacy Based on In Vitro High-Content Analysis
Source: PLoS One. 2011 Nov 2;6(11):e26230. doi: 10.1371/journal.pone.0026230 (PMC3206809; doi:10.1371/journal.pone.0026230)
Supplement: Table S3 — List of references for the 10 markers of fibrosis. (DOC) [file pone.0026230.s006.doc]

| DHE | DHE is a fluorescent dye for superoxide. Superoxide induces caspase 3-dependent apoptosis in activated HSC, but not in quiescent HSC . |
| --- | --- |
| pCREB | The nuclear transcription factor CREB is phosphorylated in the presence of elevated intracellular cAMP. Phosphorylated CREB induces target gene expression, which inhibits HSC proliferation . |
| Smad3 | Smad3 antibody staining is used to detect the level of total Smad3 in HSC. Smad 3 is in the downstream signaling pathway of TGF-*β* and is involved in the fibrogenesis process . |
| F-actin | Phalloidin dye binds to F-actin. It has been used to study adhesion and contractility of HSC . |
| BrdU | BrdU dye can be incorporated into newly synthesized DNA of replicating cells, hence it is commonly used to study cell proliferation . |
| Caspase 3 | Caspase 3 antibody staining is used to study caspase 3-dependent apoptosis of HSC . |
| ΔΨm | Mitotracker Red is used to detect the level of ΔΨm in HSC. Decrease in ΔΨm induces apoptosis . |
| Collagen III | Collagen III antibody staining is used to detect the level of collagen α1 type III in HSC. Collagen type III increases about 4 folds in a fibrotic liver . |
| MMP-2 | MMP-2 antibody staining is used to detect the level of MMP-2 (whole molecule) in HSC. The expression profile of MMP-2 changes with the fibrotic state . |
| TIMP-1 | TIMP-1 antibody staining is used to detect the level of TIMP-1 in HSC. The expression profile of TIMP-1 changes with the fibrotic state . |

Table S3. List of references for the 10 markers of fibrosis.

**References**

1. Jameel NM, Thirunavukkarasu C, Wu T, Watkins SC, Friedman SL, et al. (2009) p38-MAPK- and caspase-3-mediated superoxide-induced apoptosis of rat hepatic stellate cells: reversal by retinoic acid. J Cell Physiol 218: 157-166.

2. Mann J, Mann DA (2009) Transcriptional regulation of hepatic stellate cells. Adv Drug Deliv Rev 61: 497-512.

3. Moro T, Shimoyama Y, Kushida M, Hong YY, Nakao S, et al. (2008) Glycyrrhizin and its metabolite inhibit Smad3-mediated type I collagen gene transcription and suppress experimental murine liver fibrosis. Life Sci 83: 531-539.

4. Atorrasagasti C, Aquino JB, Hofman L, Alaniz L, Malvicini M, et al. (2011) SPARC down-regulation attenuates the profibrogenic response of hepatic stellate cells induced by TGF-{beta}1 and PDGF. Am J Physiol Gastrointest Liver Physiol.

5. Svegliati-Baroni G, Ridolfi F, Di Sario A, Casini A, Marucci L, et al. (1999) Insulin and insulin-like growth factor-1 stimulate proliferation and type I collagen accumulation by human hepatic stellate cells: differential effects on signal transduction pathways. Hepatology 29: 1743-1751.

6. Wang X, Ikejima K, Kon K, Arai K, Aoyama T, et al. (2010) Ursolic acid ameliorates hepatic fibrosis in the rat by specific induction of apoptosis in hepatic stellate cells. J Hepatol.

7. Kweon YO, Paik YH, Schnabl B, Qian T, Lemasters JJ, et al. (2003) Gliotoxin-mediated apoptosis of activated human hepatic stellate cells. J Hepatol 39: 38-46.

8. Gressner AM, Weiskirchen R (2006) Modern pathogenetic concepts of liver fibrosis suggest stellate cells and TGF-beta as major players and therapeutic targets. J Cell Mol Med 10: 76-99.

9. Hemmann S, Graf J, Roderfeld M, Roeb E (2007) Expression of MMPs and TIMPs in liver fibrosis - a systematic review with special emphasis on anti-fibrotic strategies. J Hepatol 46: 955-975.
