# Supplementary material for: Predicting In Vivo Anti-Hepatofibrotic Drug Efficacy Based on In Vitro High-Content Analysis
Source: PLoS One. 2011 Nov 2;6(11):e26230. doi: 10.1371/journal.pone.0026230 (PMC3206809; doi:10.1371/journal.pone.0026230)
Supplement: Table S4 — List of papers with pathologist-graded histological scores on fibrotic rats from 1986 to 2009. (DOC) [file pone.0026230.s007.doc]

|  | **Authors** | **Year** | **Drug(s)** | **Rat fibrotic model** | **Treatment (T) /Preventive (P)** |
| --- | --- | --- | --- | --- | --- |
| 1 | Jeong, et al. | 2005 | silymarin | CCl_4_ | P |
| 2 | Hsu, et al. | 2007 | tetrandrine, silymarin | DMN | T |
| 3 | Chong, et al. | 2006 | thalidomide, silymarin | DMN | T |
| 4 | Shu, et al. | 2009 | curcumin, colchicine | CCl_4_ | P |
| 5 | Dumont, et al. | 1986 | malotilate | CCl_4_ | P, T |
| 6 | Wu, et al. | 2008 | oxymatrine | CCl_4_ | P |
| 7 | Deng, et al. | 2009 | oxymatrine, colchicine | CCl_4_ | P |
| 8 | Seung, et al. | 2004 | colchicine | DMN | T |
| 9 | Yuan, et al. | 2004 | pioglitazone | CCl_4_ | P, T |
| 10 | Dekel, et al. | 2003 | gliotoxin | TAA | T |
| 11 | Zhen, et al. | 2007 | EGCG | CCl_4_ | P |
| 12 | Li, et al. | 2009 | rosmarinic acid, silymarin | CCl_4_ | T |
| 13 | Oakley, et al. | 2005 | sulfasalazine | CCl_4_ | T |
| 14 | Wang, et al. | 2005 | melatonin | CCl_4_ | P |
| 15 | Hong, et al. | 2009 | melatonin | CCl_4_ | P |
| 16 | Tasci, et al. | 2007 | taurine | CCl_4_ | T |
| 17 | Raetsch, et al. | 2002 | pentoxifylline | BDL | P, T |
| 18 | Marek, et al. | 2005 | PCN | CCl_4_ | P, T |
| 19 | Bruck, et al. | 2007 | curcumin | TAA | P, T |
| 20 | Liu, et al. | 2009 | astragoloside IV, colchicine | pig serum | P |
| 21 | Kuzu, et al. | 2007 | genistein | CCl_4_ | P |
| 22 | Baur, et al. | 2006 | resveratrol | high calories diet | P |
| 23 | Lv, et al. | 2007 | thalidomide | CCl_4_ | T |
| 24 | Iseri, et al. | 2007 | simvastatin | cisplatin | P |
| 25 | Lv, et al. | 2006 | thalidomide | CCl_4_ | P |
| 26 | Yeh, et al. | 2004 | thalidomide | TAA | T |
| 27 | Ryhanen, et al. | 1996 | malotilate | DMN | P |
| 28 | Tasci, et al. | 2008 | taurine | CCl_4_ | P |

Table S4. List of papers with pathologist-graded histological scores on fibrotic rats from 1986 to 2009.

**References**

1. Jeong DH, Lee GP, Jeong WI, Do SH, Yang HJ, et al. (2005) Alterations of mast cells and TGF-beta1 on the silymarin treatment for CCl(4)-induced hepatic fibrosis. World J Gastroenterol 11: 1141-1148.

2. Hsu YC, Chiu YT, Cheng CC, Wu CF, Lin YL, et al. (2007) Antifibrotic effects of tetrandrine on hepatic stellate cells and rats with liver fibrosis. J Gastroenterol Hepatol 22: 99-111.

3. Chong LW, Hsu YC, Chiu YT, Yang KC, Huang YT (2006) Anti-fibrotic effects of thalidomide on hepatic stellate cells and dimethylnitrosamine-intoxicated rats. J Biomed Sci 13: 403-418.

4. Shu JC, He YJ, Lv X, Ye GR, Wang LX (2009) Curcumin prevents liver fibrosis by inducing apoptosis and suppressing activation of hepatic stellate cells. J Nat Med 63: 415-420.

5. Dumont JM, Maignan MF, Janin B, Herbage D, Perrissoud D (1986) Effect of malotilate on chronic liver injury induced by carbon tetrachloride in the rat. J Hepatol 3: 260-268.

6. Wu XL, Zeng WZ, Jiang MD, Qin JP, Xu H (2008) Effect of Oxymatrine on the TGFbeta-Smad signaling pathway in rats with CCl4-induced hepatic fibrosis. World J Gastroenterol 14: 2100-2105.

7. Deng ZY, Li J, Jin Y, Chen XL, Lu XW (2009) Effect of oxymatrine on the p38 mitogen-activated protein kinases signalling pathway in rats with CCl4 induced hepatic fibrosis. Chin Med J (Engl) 122: 1449-1454.

8. Lee SJ, Kim YG, Kang KW, Kim CW, Kim SG (2004) Effects of colchicine on liver functions of cirrhotic rats: beneficial effects result from stellate cell inactivation and inhibition of TGF beta1 expression. Chem Biol Interact 147: 9-21.

9. Yuan GJ, Zhang ML, Gong ZJ (2004) Effects of PPARg agonist pioglitazone on rat hepatic fibrosis. World J Gastroenterol 10: 1047-1051.

10. Dekel R, Zvibel I, Brill S, Brazovsky E, Halpern Z, et al. (2003) Gliotoxin ameliorates development of fibrosis and cirrhosis in a thioacetamide rat model. Dig Dis Sci 48: 1642-1647.

11. Zhen MC, Wang Q, Huang XH, Cao LQ, Chen XL, et al. (2007) Green tea polyphenol epigallocatechin-3-gallate inhibits oxidative damage and preventive effects on carbon tetrachloride-induced hepatic fibrosis. J Nutr Biochem 18: 795-805.

12. Li GS, Jiang WL, Tian JW, Qu GW, Zhu HB, et al. (2010) In vitro and in vivo antifibrotic effects of rosmarinic acid on experimental liver fibrosis. Phytomedicine 17: 282-288.

13. Oakley F, Meso M, Iredale JP, Green K, Marek CJ, et al. (2005) Inhibition of inhibitor of kappaB kinases stimulates hepatic stellate cell apoptosis and accelerated recovery from rat liver fibrosis. Gastroenterology 128: 108-120.

14. Wang H, Wei W, Wang NP, Gui SY, Wu L, et al. (2005) Melatonin ameliorates carbon tetrachloride-induced hepatic fibrogenesis in rats via inhibition of oxidative stress. Life Sci 77: 1902-1915.

15. Hong RT, Xu JM, Mei Q (2009) Melatonin ameliorates experimental hepatic fibrosis induced by carbon tetrachloride in rats. World J Gastroenterol 15: 1452-1458.

16. Tasci I, Mas MR, Vural SA, Deveci S, Comert B, et al. (2007) Pegylated interferon-alpha plus taurine in treatment of rat liver fibrosis. World J Gastroenterol 13: 3237-3244.

17. Raetsch C, Jia JD, Boigk G, Bauer M, Hahn EG, et al. (2002) Pentoxifylline downregulates profibrogenic cytokines and procollagen I expression in rat secondary biliary fibrosis. Gut 50: 241-247.

18. Marek CJ, Tucker SJ, Konstantinou DK, Elrick LJ, Haefner D, et al. (2005) Pregnenolone-16alpha-carbonitrile inhibits rodent liver fibrogenesis via PXR (pregnane X receptor)-dependent and PXR-independent mechanisms. Biochem J 387: 601-608.

19. Bruck R, Ashkenazi M, Weiss S, Goldiner I, Shapiro H, et al. (2007) Prevention of liver cirrhosis in rats by curcumin. Liver Int 27: 373-383.

20. Liu H, Wei W, Sun WY, Li X (2009) Protective effects of astragaloside IV on porcine-serum-induced hepatic fibrosis in rats and in vitro effects on hepatic stellate cells. J Ethnopharmacol 122: 502-508.

21. Kuzu N, Metin K, Dagli AF, Akdemir F, Orhan C, et al. (2007) Protective role of genistein in acute liver damage induced by carbon tetrachloride. Mediators Inflamm 2007: 36381.

22. Baur JA, Pearson KJ, Price NL, Jamieson HA, Lerin C, et al. (2006) Resveratrol improves health and survival of mice on a high-calorie diet. Nature 444: 337-342.

23. Lv P, Luo HS, Zhou XP, Xiao YJ, Paul SC, et al. (2007) Reversal effect of thalidomide on established hepatic cirrhosis in rats via inhibition of nuclear factor-kappaB/inhibitor of nuclear factor-kappaB pathway. Arch Med Res 38: 15-27.

24. Iseri S, Ercan F, Gedik N, Yuksel M, Alican I (2007) Simvastatin attenuates cisplatin-induced kidney and liver damage in rats. Toxicology 230: 256-264.

25. Lv P, Luo HS, Zhou XP, Chireyath Paul S, Xiao YJ, et al. (2006) Thalidomide prevents rat liver cirrhosis via inhibition of oxidative stress. Pathol Res Pract 202: 777-788.

26. Yeh TS, Ho YP, Huang SF, Yeh JN, Jan YY, et al. (2004) Thalidomide salvages lethal hepatic necroinflammation and accelerates recovery from cirrhosis in rats. J Hepatol 41: 606-612.

27. Ryhanen L, Stenback F, Ala-Kokko L, Savolainen ER (1996) The effect of malotilate on type III and type IV collagen, laminin and fibronectin metabolism in dimethylnitrosamine-induced liver fibrosis in the rat. J Hepatol 24: 238-245.

28. Tasci I, Mas N, Mas MR, Tuncer M, Comert B (2008) Ultrastructural changes in hepatocytes after taurine treatment in CCl4 induced liver injury. World J Gastroenterol 14: 4897-4902.
