# Supplementary material for: Predicting In Vivo Anti-Hepatofibrotic Drug Efficacy Based on In Vitro High-Content Analysis
Source: PLoS One. 2011 Nov 2;6(11):e26230. doi: 10.1371/journal.pone.0026230 (PMC3206809; doi:10.1371/journal.pone.0026230)
Supplement: Table S5 — Mechanisms of action of the drugs. All 49 drugs are classified based on their mechanisms of action from the literature. (DOC) [file pone.0026230.s008.doc]

| **Hepatic stellate cells** | **Collagen synthesis** | **Cytokines/Receptors** | **Anti-oxidants** | **Control** |
| --- | --- | --- | --- | --- |
| lovastatin | lovastatin | matrine | taurine | paclitaxel |
| simvastatin | simvastatin | oxymatrine | olmesartan medoxomil | rotenone |
| pentoxifylline | pentoxifylline | olmesartan medoxomil | curcumin | aphidicolin |
| matrine | oxymatrine | telmisartan | resveratrol | nocodazole |
| taurine | glycyrrhizin | curcumin | genistein |  |
| PCN | olmesartan medoxomil | tetrandrine | EGCG |  |
| gliotoxin | EGCG | EGCG | silybin |  |
| olmesartan medoxomil | pirfenidone | AG1296 | silymarin |  |
| telmisartan | astragaloside IV | AG1295 | taxifolin |  |
| pioglitazone | minoxidil | PTK/ZK | melatonin |  |
| curcumin | minoxidil sulphate | imatinib mesylate |  |  |
| bortezomib | colchicine | genistein |  |  |
| MG132 | malotilate | pirfenidone |  |  |
| tetrandrine | somatostatin | camostat mesilate |  |  |
| resveratrol |  | tranilast |  |  |
| genistein |  | rosmarinic acid |  |  |
| staurosporine |  | TGF-*β* inhibitor III |  |  |
| Y27632 |  | TGF-*β* inhibitor V |  |  |
| EGCG |  | astragaloside IV |  |  |
| fasudil hydrochloride |  | colchicine |  |  |
| berberine chlroride |  |  |  |  |
| sulfasalazine |  |  |  |  |
| pirfenidone |  |  |  |  |
| rosmarinic acid |  |  |  |  |
| thalidomide |  |  |  |  |
| colchicine |  |  |  |  |
| somatostatin |  |  |  |  |

Table S5. Mechanisms of action of drugs. All 49 drugs are classified based on their mechanisms of action from the literature.

**References**

1. Rombouts K, Kisanga E, Hellemans K, Wielant A, Schuppan D, et al. (2003) Effect of HMG-CoA reductase inhibitors on proliferation and protein synthesis by rat hepatic stellate cells. J Hepatol 38: 564-572.

2. Zhang JP, Zhang M, Jin C, Zhou B, Xie WF, et al. (2001) Matrine inhibits production and actions of fibrogenic cytokines released by mouse peritoneal macrophages. Acta Pharmacol Sin 22: 765-768.

3. Miyazaki T, Karube M, Matsuzaki Y, Ikegami T, Doy M, et al. (2005) Taurine inhibits oxidative damage and prevents fibrosis in carbon tetrachloride-induced hepatic fibrosis. J Hepatol 43: 117-125.

4. Wu XL, Zeng WZ, Jiang MD, Qin JP, Xu H (2008) Effect of Oxymatrine on the TGFbeta-Smad signaling pathway in rats with CCl4-induced hepatic fibrosis. World J Gastroenterol 14: 2100-2105.

5. Kurita S, Takamura T, Ota T, Matsuzawa-Nagata N, Kita Y, et al. (2008) Olmesartan ameliorates a dietary rat model of non-alcoholic steatohepatitis through its pleiotropic effects. Eur J Pharmacol 588: 316-324.

6. Toda K, Kumagai N, Kaneko F, Tsunematsu S, Tsuchimoto K, et al. (2009) Pentoxifylline prevents pig serum-induced rat liver fibrosis by inhibiting interleukin-6 production. J Gastroenterol Hepatol 24: 860-865.

7. Hernandez E, Correa A, Bucio L, Souza V, Kershenobich D, et al. (2002) Pentoxifylline diminished acetaldehyde-induced collagen production in hepatic stellate cells by decreasing interleukin-6 expression. Pharmacol Res 46: 435-443.

8. Kurikawa N, Suga M, Kuroda S, Yamada K, Ishikawa H (2003) An angiotensin II type 1 receptor antagonist, olmesartan medoxomil, improves experimental liver fibrosis by suppression of proliferation and collagen synthesis in activated hepatic stellate cells. Br J Pharmacol 139: 1085-1094.

9. Lin J, Zheng S, Chen A (2009) Curcumin attenuates the effects of insulin on stimulating hepatic stellate cell activation by interrupting insulin signaling and attenuating oxidative stress. Lab Invest 89: 1397-1409.

10. Zhang JP, Zhang M, Zhou JP, Liu FT, Zhou B, et al. (2001) Antifibrotic effects of matrine on in vitro and in vivo models of liver fibrosis in rats. Acta Pharmacol Sin 22: 183-186.

11. Shi GF, Li Q (2005) Effects of oxymatrine on experimental hepatic fibrosis and its mechanism in vivo. World J Gastroenterol 11: 268-271.

12. Jin H, Yamamoto N, Uchida K, Terai S, Sakaida I (2007) Telmisartan prevents hepatic fibrosis and enzyme-altered lesions in liver cirrhosis rat induced by a choline-deficient L-amino acid-defined diet. Biochem Biophys Res Commun 364: 801-807.

13. Kawada N, Seki S, Inoue M, Kuroki T (1998) Effect of antioxidants, resveratrol, quercetin, and N-acetylcysteine, on the functions of cultured rat hepatic stellate cells and Kupffer cells. Hepatology 27: 1265-1274.

14. Chen YX, Zhang XR, Xie WF, Li S (2004) Effects of taurine on proliferation and apoptosis of hepatic stellate cells in vitro. Hepatobiliary Pancreat Dis Int 3: 106-109.

15. Moro T, Shimoyama Y, Kushida M, Hong YY, Nakao S, et al. (2008) Glycyrrhizin and its metabolite inhibit Smad3-mediated type I collagen gene transcription and suppress experimental murine liver fibrosis. Life Sci 83: 531-539.

16. Lin J, Chen A (2008) Activation of peroxisome proliferator-activated receptor-gamma by curcumin blocks the signaling pathways for PDGF and EGF in hepatic stellate cells. Lab Invest 88: 529-540.

17. Shi HY, Xu JW, Ren XX (2008) [Effect of genistein on hepatic stellate cell proliferation and lipid peroxidation in vitro]. Nan Fang Yi Ke Da Xue Xue Bao 28: 2066-2068.

18. Marek CJ, Wallace K, Durward E, Koruth M, Leel V, et al. (2009) Low affinity glucocorticoid binding site ligands as potential anti-fibrogenics. Comp Hepatol 8: 1.

19. Chen YW, Li DG, Wu JX, Lu HM (2005) Tetrandrine inhibits activation of rat hepatic stellate cells stimulated by transforming growth factor-beta in vitro via up-regulation of Smad 7. J Ethnopharmacol 100: 299-305.

20. Fu Y, Zheng S, Lu SC, Chen A (2008) Epigallocatechin-3-gallate inhibits growth of activated hepatic stellate cells by enhancing the capacity of glutathione synthesis. Mol Pharmacol 73: 1465-1473.

21. Wright MC, Issa R, Smart DE, Trim N, Murray GI, et al. (2001) Gliotoxin stimulates the apoptosis of human and rat hepatic stellate cells and enhances the resolution of liver fibrosis in rats. Gastroenterology 121: 685-698.

22. Nakamuta M, Higashi N, Kohjima M, Fukushima M, Ohta S, et al. (2005) Epigallocatechin-3-gallate, a polyphenol component of green tea, suppresses both collagen production and collagenase activity in hepatic stellate cells. Int J Mol Med 16: 677-681.

23. Yasuda Y, Shimizu M, Sakai H, Iwasa J, Kubota M, et al. (2009) (-)-Epigallocatechin gallate prevents carbon tetrachloride-induced rat hepatic fibrosis by inhibiting the expression of the PDGFRbeta and IGF-1R. Chem Biol Interact 182: 159-164.

24. Trappoliere M, Caligiuri A, Schmid M, Bertolani C, Failli P, et al. (2009) Silybin, a component of sylimarin, exerts anti-inflammatory and anti-fibrogenic effects on human hepatic stellate cells. J Hepatol 50: 1102-1111.

25. Tada S, Nakamuta M, Enjoji M, Sugimoto R, Iwamoto H, et al. (2001) Pirfenidone inhibits dimethylnitrosamine-induced hepatic fibrosis in rats. Clin Exp Pharmacol Physiol 28: 522-527.

26. Melton AC, Yee HF (2007) Hepatic stellate cell protrusions couple platelet-derived growth factor-BB to chemotaxis. Hepatology 45: 1446-1453.

27. Li JT, Liao ZX, Ping J, Xu D, Wang H (2008) Molecular mechanism of hepatic stellate cell activation and antifibrotic therapeutic strategies. J Gastroenterol 43: 419-428.

28. Li SP, Xu XY, Sun Z, Chen Z (2007) [Astragalus polysaccharides and astragalosides regulate cytokine secretion in LX-2 cell line]. Zhejiang Da Xue Xue Bao Yi Xue Ban 36: 543-548.

29. Iwamoto H, Nakamuta M, Tada S, Sugimoto R, Enjoji M, et al. (2000) Platelet-derived growth factor receptor tyrosine kinase inhibitor AG1295 attenuates rat hepatic stellate cell growth. J Lab Clin Med 135: 406-412.

30. Vitaglione P, Morisco F, Caporaso N, Fogliano V (2004) Dietary antioxidant compounds and liver health. Crit Rev Food Sci Nutr 44: 575-586.

31. Kawaguchi K, Sakaida I, Tsuchiya M, Omori K, Takami T, et al. (2004) Pioglitazone prevents hepatic steatosis, fibrosis, and enzyme-altered lesions in rat liver cirrhosis induced by a choline-deficient L-amino acid-defined diet. Biochem Biophys Res Commun 315: 187-195.

32. Parish JL, Hughes MA, Cherry GW, Ferguson DJ (1995) The effect of minoxidil analogues and metabolites on the contraction of collagen lattices by human skin fibroblasts. Br J Plast Surg 48: 154-160.

33. Liu Y, Wen XM, Lui EL, Friedman SL, Cui W, et al. (2009) Therapeutic targeting of the PDGF and TGF-beta-signaling pathways in hepatic stellate cells by PTK787/ZK22258. Lab Invest 89: 1152-1160.

34. Padillo FJ, Cruz A, Navarrete C, Bujalance I, Briceno J, et al. (2004) Melatonin prevents oxidative stress and hepatocyte cell death induced by experimental cholestasis. Free Radic Res 38: 697-704.

35. Zheng S, Chen A (2004) Activation of PPARgamma is required for curcumin to induce apoptosis and to inhibit the expression of extracellular matrix genes in hepatic stellate cells in vitro. Biochem J 384: 149-157.

36. Yoshiji H, Noguchi R, Kuriyama S, Ikenaka Y, Yoshii J, et al. (2005) Imatinib mesylate (STI-571) attenuates liver fibrosis development in rats. Am J Physiol Gastrointest Liver Physiol 288: G907-913.

37. Anan A, Baskin-Bey ES, Bronk SF, Werneburg NW, Shah VH, et al. (2006) Proteasome inhibition induces hepatic stellate cell apoptosis. Hepatology 43: 335-344.

38. Jiang J, Zhong C, Yu Y, Zhang Q (1996) Colchicine reduces hepatic fibrosis in mice infected with Schistosoma japonicum. Chin Med J (Engl) 109: 795-800.

39. Qi LH, Kang LP, Zhang JP, Shi N, Zhang M, et al. (2001) [Antifibrotic effects of genistein and quercetin in vitro]. Yao Xue Xue Bao 36: 648-651.

40. Ala-Kokko L, Stenback F, Ryhanen L (1987) Preventive effect of malotilate on carbon tetrachloride-induced liver damage and collagen accumulation in the rat. Biochem J 246: 503-509.

41. Di Sario A, Bendia E, Macarri G, Candelaresi C, Taffetani S, et al. (2004) The anti-fibrotic effect of pirfenidone in rat liver fibrosis is mediated by downregulation of procollagen alpha1(I), TIMP-1 and MMP-2. Dig Liver Dis 36: 744-751.

42. Yin MF, Lian LH, Piao DM, Nan JX (2007) Tetrandrine stimulates the apoptosis of hepatic stellate cells and ameliorates development of fibrosis in a thioacetamide rat model. World J Gastroenterol 13: 1214-1220.

43. Reynaert H, Vaeyens F, Qin H, Hellemans K, Chatterjee N, et al. (2001) Somatostatin suppresses endothelin-1-induced rat hepatic stellate cell contraction via somatostatin receptor subtype 1. Gastroenterology 121: 915-930.

44. Okuno M, Akita K, Moriwaki H, Kawada N, Ikeda K, et al. (2001) Prevention of rat hepatic fibrosis by the protease inhibitor, camostat mesilate, via reduced generation of active TGF-beta. Gastroenterology 120: 1784-1800.

45. Souza IC, Martins LA, Coelho BP, Grivicich I, Guaragna RM, et al. (2008) Resveratrol inhibits cell growth by inducing cell cycle arrest in activated hepatic stellate cells. Mol Cell Biochem 315: 1-7.

46. Ikeda H, Inao M, Fujiwara K (1996) Inhibitory effect of tranilast on activation and transforming growth factor beta 1 expression in cultured rat stellate cells. Biochem Biophys Res Commun 227: 322-327.

47. McCarty MF, Barroso-Aranda J, Contreras F (2009) Genistein and phycocyanobilin may prevent hepatic fibrosis by suppressing proliferation and activation of hepatic stellate cells. Med Hypotheses 72: 330-332.

48. Li GS, Jiang WL, Tian JW, Qu GW, Zhu HB, et al. In vitro and in vivo antifibrotic effects of rosmarinic acid on experimental liver fibrosis. Phytomedicine 17: 282-288.

49. Ramm GA, Li L, Britton RS, O'Neill R, Bacon BR (2003) Effect of protein kinase C activation and inhibition on rat hepatic stellate cell activation. Dig Dis Sci 48: 790-796.

50. Iwamoto H, Nakamuta M, Tada S, Sugimoto R, Enjoji M, et al. (2000) A p160ROCK-specific inhibitor, Y-27632, attenuates rat hepatic stellate cell growth. J Hepatol 32: 762-770.

51. Higashi N, Kohjima M, Fukushima M, Ohta S, Kotoh K, et al. (2005) Epigallocatechin-3-gallate, a green-tea polyphenol, suppresses Rho signaling in TWNT-4 human hepatic stellate cells. J Lab Clin Med 145: 316-322.

52. Wang YZ, Jiang HQ, Hu CX, Chen X (2006) [Fasudil inhibits HSC adhesion, migration and proliferation via Rho/ROCK pathway]. Zhonghua Gan Zang Bing Za Zhi 14: 821-823.

53. Lee SJ, Kim YG, Kang KW, Kim CW, Kim SG (2004) Effects of colchicine on liver functions of cirrhotic rats: beneficial effects result from stellate cell inactivation and inhibition of TGF beta1 expression. Chem Biol Interact 147: 9-21.

54. Sun X, Zhang X, Hu H, Lu Y, Chen J, et al. (2009) Berberine inhibits hepatic stellate cell proliferation and prevents experimental liver fibrosis. Biol Pharm Bull 32: 1533-1537.

55. Oakley F, Meso M, Iredale JP, Green K, Marek CJ, et al. (2005) Inhibition of inhibitor of kappaB kinases stimulates hepatic stellate cell apoptosis and accelerated recovery from rat liver fibrosis. Gastroenterology 128: 108-120.

56. Zhao XY, Zeng X, Li XM, Wang TL, Wang BE (2009) Pirfenidone inhibits carbon tetrachloride- and albumin complex-induced liver fibrosis in rodents by preventing activation of hepatic stellate cells. Clin Exp Pharmacol Physiol 36: 963-968.

57. Paul SC, Lv P, Xiao YJ, An P, Liu SQ, et al. (2006) Thalidomide in rat liver cirrhosis: blockade of tumor necrosis factor-alpha via inhibition of degradation of an inhibitor of nuclear factor-kappaB. Pathobiology 73: 82-92.

58. Pan Q, Li DG, Lu HM, Lu LY, Wang YQ, et al. (2004) Antiproliferative and proapoptotic effects of somatostatin on activated hepatic stellate cells. World J Gastroenterol 10: 1015-1018.
