# Supplementary material for: Predicting In Vivo Anti-Hepatofibrotic Drug Efficacy Based on In Vitro High-Content Analysis
Source: PLoS One. 2011 Nov 2;6(11):e26230. doi: 10.1371/journal.pone.0026230 (PMC3206809; doi:10.1371/journal.pone.0026230)
Supplement: Material S1 — Computing E predict from cellular feature values. (DOC) [file pone.0026230.s009.doc]

**Supplementary materials S1. Computing E*_predict_* from cellular feature values.**

Step1. Kolmogorov-Smirnov statistics for unimodal distributions:

$$KS_{mfc}=\sup_{x}\left|F_{mfc}(x)-F_{mf0}(x\left.)\right|\right.$$

where sup is the least upper bound function, and $F_{mfc}$is the empirical distribution function

$$F_{mfc}(x)=\frac{1}{N}\sum_{i=1}^{N} I_{X_{mfci}\leq x}$$

$$I=\left\{\begin{matrix} 1\text{ }if\text{ }X_{mfci}\leq x \\ 0\text{ }otherwise \end{matrix}\right.$$

where *N* is the total number of cells captured in a well with drug concentration *c* and marker *m*. $X_{mfci}$ represents the value of feature *f* for cell *i* in that well.

Ratio for bimodal distributions:

$$R_{mfc}=r_{mfc}-r_{mf0}$$

$$r_{mfc}=\frac{1}{N}\sum_{i=k_{\min}}^{K} m_{mfci}$$

where *K* is the total number of bins in a histogram of feature values for feature *f* for cells captured in a well with drug concentration *c* and marker *m*. $m_{mfci}$is the number of cells in the *i*th bin, $k_{\min}$ is the bin with the minimum number of cells to the right of the first peak.

*KR* values:

$$KR_{mfc}=\left\{\begin{matrix} KS_{mfc}\text{ if }f\text{ has unimodal distribution} \\ R_{mfc}\text{ if }f\text{ has bimodal distribution} \end{matrix}\right.$$

(Higher multimodal distributions are not observed in the dataset.)

Step 2. Use trapezoidal rule to estimate the area under the curve (*KR* vs. relative drug concentration) for each feature *f* of cells stained with marker *m*:

$$AUC_{mf}=\frac{1}{2}\times\sum_{c=0}^{C} (KR_{mfc}+KR_{mf(c+1)})\times2^{c}$$

where *C* equals 10 in this study as 11 concentrations are used per drug. $c=0$ for control cells without drug treatment. 2*^c^* accounts for the two-time serial dilution that was made during drug preparation. The relative drug concentration for all drugs ranges from 2^0^ to 2^11^. Compared with *KR* values at a particular concentration, *AUC* is more resistant to noise as positive and negative fluctuations in area under the curve tend to cancel each other out, leaving a more consistent and reproducible value (data not shown).

Step 3. Compute sign corrected sum of *AUC* for each marker:

$$SAUC_{m}=\sum_{f} AUC_{mf}\times S_{mf}$$

where $S_{mf}$ is -1, 0 or 1. If a feature value decreases under the influence of anti-fibrotic drugs (e.g. ΔΨm drops in apoptotic cells), $S_{mf}$ is equal to -1. If the feature value increases, $S_{mf}$ is equal to 1. If the direction of change cannot be clearly determined, $S_{mf}$ is assigned to 0. Since most of the drugs in this study have been previously shown to have anti-fibrotic effects, it is assumed that feature variations in the majority of drugs will change in the anti-fibrotic direction and the $S_{mf}$ values are assigned accordingly. $AUC_{mf}\times S_{mf}$ always increases under the influence of anti-fibrotic drugs.

Nuclear staining is done for all cells. To avoid redundancy of counting nuclear channel information 10 times, the 5 nuclear channel features are only used in computing *SAUC* for BrdU stained cells.

Step 4. Computing E*_predict_:*

$$E_{predict}=\left\{\begin{matrix} 0\text{ all non-specific drugs} \\ \sum_{m} SAUC_{m}\times W_{m}^{opt}\text{ otherwise} \end{matrix}\right.$$

where $W_{m}^{opt}$is the optimized weight for marker *m* as shown in Fig. 4. $C_{highest}$ is the highest drug working concentration in supplementary Table 1. Non-specific drugs have been identified from the analysis on the variation of the percentage total collagen III intensity with drug concentrations. If a negative index value is obtained, the index will be assigned to 0 value.
