# Supplementary material for: Predicting In Vivo Anti-Hepatofibrotic Drug Efficacy Based on In Vitro High-Content Analysis
Source: PLoS One. 2011 Nov 2;6(11):e26230. doi: 10.1371/journal.pone.0026230 (PMC3206809; doi:10.1371/journal.pone.0026230)
Supplement: Material S2 — Method to identify drugs with non-specific effects from in vitro HCA analysis. (DOC) [file pone.0026230.s010.doc]

**Supplementary Materials S2. Method to identify drugs with non-specific effects from *in vitro* HCA analysis**

Drugs that target only the non-specific pathways such as proliferation and apoptosis (non-specific drugs) were eliminated to ensure that the system was specific for anti-fibrosis study. In agreement with other high-throughput anti-fibrotic systems , collagen expression level was used as an indicator to identify non-specific drugs. Since the percentage collagen (type III as an example) intensity in the control cells without drugs does not fluctuate more than 7% (p ≈ 10^-8^), any drug that caused more than 7% increase in collagen III intensity at its highest concentration was defined as a non-specific drug. A total of 49 drugs were screened. Using pioglitazone, epigallocatechin gallate (EGCG) and aphidicolin as examples, graphs of percentage collagen III intensity versus increasing drug concentrations were plotted for cells treated with each of the three drugs. A decreasing trend can be clearly seen for cells treated with pioglitazone and EGCG, which reduced collagen production by 33% and 22% respectively (supplementary Fig. 3A, B); on the other hand, the percentage value increased from 100% to 161% for aphidicolin (supplementary Fig. 3C). The standard deviations from two replicate experiments (error bars) are relatively small for most of the data points, showing the reproducibility of the HCA system. This approach identified 14 non-specific drugs from a total of 49 drugs. All 4 randomly chosen non-specific controls including aphidicolin, rotenone, paclitaxel and nocodazole were successfully identified. These drugs affect non-specific pathways like cell proliferation or apoptosis, but have not been documented to have anti-fibrosis effects.

The *in vitro* efficacies of drugs depend on their concentration and treatment. Since the primary target of many drugs is not directly affecting collagen expression, the collagen level of drug treated cells may not show a significant decrease under the experimental conditions. As a result, a relaxed condition was used as the first screening step. Drugs causing more than a 7% increase in collagen were removed from further analysis. Subsequent procedures examined the overall drug induced cellular changes. Drugs that failed to produce a significant response would be reflected by their low index values. On the other hand, if a drug showed an overall high efficacy, it could be recommended for further characterization. One such example is sulfasalazine, which at its highest concentration, did not cause a significant decrease in collagen type III expression. However, a short-term study using a single injection of sulfasalazine reduced the fibrosis score from 3.0 in CCl_4_ only rat livers to 1.5 . Our subsequent analysis also yielded a high E*_predict_* at 39437.

**References**

1. Hashem MA, Jun KY, Lee E, Lim S, Choo HY, et al. (2008) A rapid and sensitive screening system for human type I collagen with the aim of discovering potent anti-aging or anti-fibrotic compounds. Mol Cells 26: 625-630.

2. Chen CZ, Peng YX, Wang ZB, Fish PV, Kaar JL, et al. (2009) The Scar-in-a-Jar: studying potential antifibrotic compounds from the epigenetic to extracellular level in a single well. Br J Pharmacol 158: 1196-1209.

3. Oakley F, Meso M, Iredale JP, Green K, Marek CJ, et al. (2005) Inhibition of inhibitor of kappaB kinases stimulates hepatic stellate cell apoptosis and accelerated recovery from rat liver fibrosis. Gastroenterology 128: 108-120.
